# Novel Reporter for Faithful Monitoring of ERK2 Dynamics in Living Cells and Model Organisms

**DOI:** 10.1371/journal.pone.0140924

**Published:** 2015-10-30

**Authors:** François Sipieter, Benjamin Cappe, Mariano Gonzalez Pisfil, Corentin Spriet, Jean-François Bodart, Katia Cailliau-Maggio, Peter Vandenabeele, Laurent Héliot, Franck B. Riquet

**Affiliations:** 1 Molecular Signaling and Cell Death Unit, Department of Biomedical Molecular Biology, Ghent University, Ghent, Belgium; 2 Molecular Signaling and Cell Death Unit, Inflammation Research Center (IRC), VIB, Ghent, Belgium; 3 Methusalem Program, Ghent University, Ghent, Belgium; 4 Equipe Biophotonique Cellulaire Fonctionnelle, Laboratoire de Physique des Lasers, Atomes et Molécules (PhLAM), CNRS-UMR 8523, Villeneuve d'Ascq, France; 5 Regulation of Signal Division Team, Structural and Functional Glycobiology Unit (UGSF), CNRS-UMR 8576, Lille 1 University, Villeneuve d’Ascq, France; 6 Structural and Functional Glycobiology Unit (UGSF), CNRS-UMR 8576, Lille 1 University, Villeneuve d’Ascq, France; 7 TISBio, Structural and Functional Glycobiology Unit (UGSF), CNRS-UMR 8576, FR3688, Lille 1 University, Villeneuve d’Ascq, France; 8 Groupement de Recherche Microscopie Imagerie du Vivant, GDR2588 MIV-CNRS, Villeneuve d'Ascq, France; Université de Sherbrooke, CANADA

## Abstract

Uncoupling of ERK1/2 phosphorylation from subcellular localization is essential towards the understanding of molecular mechanisms that control ERK1/2-mediated cell-fate decision. ERK1/2 non-catalytic functions and discoveries of new specific anchors responsible of the subcellular compartmentalization of ERK1/2 signaling pathway have been proposed as regulation mechanisms for which dynamic monitoring of ERK1/2 localization is necessary. However, studying the spatiotemporal features of ERK2, for instance, in different cellular processes in living cells and tissues requires a tool that can faithfully report on its subcellular distribution. We developed a novel molecular tool, ERK2-LOC, based on the T2A-mediated coexpression of strictly equimolar levels of eGFP-ERK2 and MEK1, to faithfully visualize ERK2 localization patterns. MEK1 and eGFP-ERK2 were expressed reliably and functionally both *in vitro* and in single living cells. We then assessed the subcellular distribution and mobility of ERK2-LOC using fluorescence microscopy in non-stimulated conditions and after activation/inhibition of the MAPK/ERK1/2 signaling pathway. Finally, we used our coexpression system in *Xenopus laevis* embryos during the early stages of development. This is the first report on MEK1/ERK2 T2A-mediated coexpression in living embryos, and we show that there is a strong correlation between the spatiotemporal subcellular distribution of ERK2-LOC and the phosphorylation patterns of ERK1/2. Our approach can be used to study the spatiotemporal localization of ERK2 and its dynamics in a variety of processes in living cells and embryonic tissues.

## Introduction

Extracellular signal-Regulated protein Kinases 1 and 2 (ERK1/2) are members of the Mitogen Activated Protein Kinase (MAPK) superfamily. The ERK1/2 signaling pathway plays an important role in the cellular signaling network by regulating several cellular processes, such as cell survival, proliferation, migration, differentiation and death, depending on the cellular context [1,2]. The ERK1/2 signaling pathway displays the characteristic three-tiered core cascade MAPK architecture [3], ensuring not only signal transduction but also amplification of signals from different membrane-stimulated receptors, such as Receptor Tyrosine Kinases (RTK) and G Protein-Coupled Receptors (GPCRs) [4,5]. Activation of the pathway by different extracellular stimuli triggers sequential phosphorylation of the protein kinases Raf, MAPK/ERK Kinase 1/2 (MEK1/2) and ERK1/2, which constitute a conserved signaling module. Compelling evidence indicates that the ERK1/2 cascade is involved in the pathogenesis, progression and oncogenic behavior of several human cancers, including lung, breast, colorectal and pancreatic cancer, as well as glioblastoma and melanoma [6,7].

Though the biochemical events of ERK1/2 signaling have been well characterized, a central question remains: How can this signaling cascade trigger different cellular outcomes? An increasing number of papers have shown that modulation of the duration, magnitude and subcellular compartmentalization of ERK1/2 activity by specific key regulators are interpreted by the cell to determine cell fate [8,9]. Moreover, preservation of the integrity of cell decisions requires control of the dynamic subcellular distribution of ERK1/2 and its ability to access ERK1/2 substrates. In resting cells, components of the ERK1/2 signaling pathway are mainly sequestered in the cytoplasm by cytoplasmic scaffold/anchoring proteins [10]. One of the positive regulators of the ERK1/2 cascade is the evolutionarily conserved Kinase Suppressor of Ras (KSR), which facilitates activation of the pathway by bringing the components of ERK1/2 signaling close to Ras at the plasma membrane [11]. MEK1 is sequestered in the cytoplasm of resting cells by its N-terminal nuclear export sequence (NES) and functions as a cytoplasmic anchor for inactive ERK2 [12]. Upon extracellular stimulation and activating phosphorylation, MEK1 and ERK2 are released from cytoplasmic anchors and rapidly translocate into the nucleus [13–16]. Besides its apparent cytoplasmic localization, 5% of MEK1 can be found in the nucleus at the peak of activation of the pathway [17]. MEK1 can rapidly transit between the cytoplasm and the nucleus much faster than ERK2 and therefore acts as a nuclear export shuttle for ERK2 and other nuclear proteins [18]. Besides differences between cells in spatiotemporal dynamics of ERK1/2 [19], it appears that ERK1/2 phosphorylation and subcellular distribution are uncoupled in several cellular models due to interaction of ERK1/2 with various anchors/scaffolds [20,21]. Upon mitogenic stimulation, ERK1/2 signaling upregulates the expression of short-lived nuclear anchors such as MAPK phosphatases (MKP), which leads to dephosphorylation of ERK1/2 and accumulation of its inactive form in the nucleus several hours after pathway activation [21,22]. Monitoring the dynamic behavior of ERK1/2 in single cells will resolve this apparently conflictual relationship and evaluate the effects of specific regulators of ERK1/2 compartmentalization on cell fate determination.

To visualize ERK1/2 dynamics in living cells, various studies used ERK1/2 tagged with GFP-like fluorescent proteins and found that overexpressed eGFP-ERK2 is predominantly localized in the nucleus of resting cells. This unexpected localization of eGFP-ERK2 was due to the disruption of MEK/ERK balance [12,15]. This problem has been often ignored [16,23–25] or tackled by coexpression of MEK1 to restore the balance and the cytoplasmic localization of ERK2 expressed at high levels in serum-starved cultures without stimulation [26,27]. These coexpression strategies mostly suffer from the inconsistency of the coexpression patterns of ERK2 and MEK1 in different cells. Coexpression of eGFP-ERK2 and MEK1 is generally associated with an abnormally short persistence of eGFP-ERK2 in the nucleus of resting cells, in contrast to endogenous ERK2, which remains in the nucleus several hours after mitogenic stimulation [21,26,27]. To overcome this difficulty, other studies selected cells expressing low levels of eGFP-ERK2 (100–150 nM) compared to the estimated endogenous protein level (1 μM) [28] to obtain a faithful localization profile of the kinase in serum-starved conditions [29]. However, transfected cells are dimly fluorescent, which are unsuitable for long-term video imaging. As a new approach to maintain the endogenous MEK/ERK balance, an exogenous tagged version of ERK1 was re-expressed in ERK1-deficient cell lines by transient transfection of a plasmid encoding ERK1 under the control of a strong promoter [30]. Cells were selected for tagged-ERK1 expression level on the basis that the nucleus was not brighter than the cytoplasm in the starved conditions. Nevertheless, the delicate MEK/ERK balance was progressively disrupted a few hours after transfection, resulting again in aberrant nuclear accumulation of ERK1 in non-stimulated conditions. Another study used a retroviral tagging approach and introduced the full-length sequence of YFP as a new exon into one allele of the *erk2* gene [19]. The tagged ERK2 was in minority compared to the wild-type protein, which led to proper subcellular distribution of tagged-ERK2 in the starved conditions. But again the fluorescent intensity was dim due to the low expression level. All these approaches are limited by the need for severe imaging conditions (causing phototoxicity, photobleaching and decrease of signal to noise ratio) that are not compatible with live cell video-microscopy, especially considering the stress-sensitive nature of MAPK pathways [31].

To avoid the artefacts in ERK2 localization patterns and facilitate the long-term functional imaging, we developed a novel ERK2 localization reporter named ERK2-LOC. We employed the T2A-mediated coexpression of ERK2 and MEK1 to enable faithful monitoring of eGFP-ERK2 localization dynamics in both basal and growth factor-stimulated conditions. Our procedure was characterized using standard biochemical approaches and validated by live-cell imaging in living NIH-3T3 cells. Final verification was conducted in the *Xenopus laevis* model during the early developmental stages. This is the first time that ERK2 localization is studied in living embryos. Our simple approach can be used for the reliable study of the spatiotemporal dynamic of ERK2 in living cells and in live model organisms.

## Materials and Methods

### Ethics Statement

All animal experiments were performed at Lille 1 University according to the rules of the European Community Council guidelines (86/609/EEC) for laboratory animal experimentation. The local institutional review board (*Comité d’Ethique en Expérimentation Animale Nord-Pas-De-Calais* (CEEA, 07/2010) approved all animal experimental protocols in this study.

### Reagents

Recombinant mouse fibroblast growth factor 4 (FGF4, #5846-F4-025/CF) was purchased from R&D Systems and fetal bovine serum (FBS, #10082–147) from Gibco, Life Technologies. Other reagents, *e*.*g*., bovine serum albumin (BSA, fraction V, #05482), dimethylsulfoxide (DMSO, #D8418) and MEK inhibitor (U0126, #U120) were from Sigma Aldrich.

### Plasmid constructs

The plasmid pCS2-Myr-TdTomato-T2A-Histone2B-GFP was kindly provided by Dr. Shankar Srinivas (Department of Physiology Anatomy and Genetics, University of Oxford, United Kingdom). *Xenopus laevis* ERK2 (xERK2) plasmid was a kind gift from Dr. Lynn Heasley (Health Science Center, University of Colorado, Denver, USA). The plasmid encoding *Rattus norvegicus* ERK2 (rERK2) fused at its N-terminal to the enhanced green fluorescent protein (eGFP-rERK2) was a kind gift from Dr. Georges Baffet (UMR1085 INSERM, University of Rennes, France). All oligonucleotides are listed in Table 1. The synthetized DNA sequence encoding the *Thosea asigna* virus 2A peptide (T2A peptide) was inserted into the pCS2+ backbone in frame between untagged *Mus musculus* MEK1 (mMEK1) and eGFP-rERK2 to build the expression vector pCS2-mMEK1-2A-eGFP-rERK2 (abbreviated rERK2-LOC). We next fused a mCherry to the N-terminus of mMEK1 to generate the construct named pCS2-mCherry-mMEK1-2A-eGFP-rERK2. The full-length cDNA sequences of MEK1 and ERK2 from *Xenopus laevis* were subcloned upstream and downstream, respectively, of the T2A peptide. We fused an eGFP to the N-terminus of xERK2 to generate the construct named pCS2-xMEK1-2A-eGFP-xERK2 (abbreviated xERK2-LOC). As a control, we fused the T2A sequence to the N-terminus of eGFP-xERK2 (pCS2-2A-eGFP-xERK2). Based on published studies, we kept a Gly-Ser-Gly (GSG) linker between MEK1 and the T2A sequences to optimize cleavage efficiency [32,33]. The cloning procedure is detailed in S1 File. All PCR products were gel purified and digested with restriction endonucleases according to the cloning strategies. All resulting constructs were verified by restriction digestion followed by agarose gel electrophoresis, or by PCR colony screening (#2200210, MasterTaq Kit, 5Prime), and then validated by sequencing (Genoscreen, France). Restriction endonucleases *Pfu* and *Taq* DNA polymerase, Klenow fragment, Mung Bean Nuclease, T4 Polynucleotide kinase (PNK), T4 DNA ligase, as well as dNTPs, ATP and specific buffers were purchased from New England Biolabs. All oligonucleotides were synthesized by Eurogentech (Belgium). Each complementary oligonucleotide designed to create double-stranded cassettes was 5’-phosphorylated by T4 PNK and then purified using Bio-Gel P-6 Micro Bio-Spin chromatography columns (#732–6222, Biorad). DNA fragments were all purified on Qiagen plasmid purification columns (#28106, #28706 and # 27106, Qiagen).

**Table 1:** Sequence of oligonucleotide primers used in this study.

Primer name	Oligonucleotide sequence (5’→3’)	Tm(°C) / %GC
BackboneΔAgeI-F	GCTACTTGTTCTTTTTGCA ACCGGT GGATCCCATCGATTCGAATTC	70 / 46
Backbone7G-F1	CCGGT GGCGCGCC GCTAGC GGTGGCGGAGGTGGCGGAGGTTA	84 / 76
Backbone7G-R1	CCGGTAACCTCCGCCACCTCCGCCACC GCTAGC GGCGCGCC A	84 / 76
Backbone7G-F2	CTAGCGGC ACCGGT GGC TGTACA AGGGAGGCGGTGGAGGCGGTGGG	82 / 72
Backbone7G-R2	CTAGCCCACCGCCTCCACCGCCTCCCT TGTACA GCC ACCGGT GCCG	82 / 72
Backbone7G-F3	CCGGAGGTGGCGGAGGTGGCGGG ACTAGT CCA GGCGCGCC TCCGC	84 / 78
Backbone7G-R3	TCGAGCGGA GGCGCGCC TGG ACTAGT CCCGCCACCTCCGCCACCT	84 / 73
xERK2.AgeI-BamHI-F	T ACCGGT GGATCC AC **ATG** GCAGCGGCAGCGGCCTCGTC	79 / 68
xERK2.XhoI-R	GAGG CTCGAG **TCA** GTACCCTGGCTGGAATCTAGCG	71 / 60
eGFP.AscI-F	CGCC GGCGCGCC AGCCATGGTGAGCAAGGGCGAGG	81 / 77
eGFP.NheI-R	ACC GCTAGC CTTGTACAGCTCGTCCATGCC	70 / 60
T2A.AscI-F	CGCGCCGG ACTAGT CC ATCGAT GGCAGTGGAGAGGGCAGAGGAAGTCTGCTAACATGCGGTGACGTCGAGGAGAATCCTGGCCCAGGTGG	84 / 62
T2A.AscI-R	CGCGCCACCTGGGCCAGGATTCTCCTCGACGTCACCGCATGTTAGCAGACTTCCTCTGCCCTCTCCACTGCC ATCGAT GG ACTAGT CCGG	84 / 62
xMEK1.SpeI-F	GG ACTAGT CCAACATGCCTAAAAAGAAGCCT	64 / 45
xMEK1.ClaI-F	CC ATCGAT GGCCACTCCGGCGGCATGGGTTG	74 / 68
mMEK1.SpeI-F1	GG ACTAGT CCAAGATGCCCAAGAAGAAGCCG	67 / 55
mMEK1.SpeI-F2	GG ACTAGT CCCAAGAAGAAGCCGACGCCCATCCAGCTG	73 / 61
mMEK1.ClaI-R	CC ATCGAT GGCGATGCTGG CAGCGTGGGTTG	73 / 65
mMEK1.AscI-R	A GGCGCGCC **TCA** GATGCTGGCAGCGTGGGTTGGTGTGCTGGG	81/ 69

Abbreviations: 7G, 7-glycine linker; F, forward primer; R, reverse primer. Restriction enzyme sites are underlined and start/stop codons are in bold.

### Cell Culture and Transfection

NIH-3T3 cells were purchased from American Type Culture Collection (VA, USA) and maintained at 37°C under 5% CO_2_ in Dulbecco’s Modified Eagle Medium (DMEM, #11885–084) supplemented with 10% FBS and 100 U/mL penicillin/streptomycin (P/S, #15140–122) (Gibco, Life Technologies). For live imaging, NIH3T3 cells were plated on 35-mm dishes (#81156, ibiTreat, Ibidi) to reach 60% confluence at the time of transfection, performed using JetPrime reagent (#114–15, Polyplus) according to the manufacturer’s instructions. Cells were starved by adding 1% FBS for 24 h before experiments began. One hour before cell imaging, medium was replaced with preheated Leibovitz L-15 bicarbonate-free medium (#11415–049, Gibco, Life Technologies) supplemented with 1% FBS and 100 units/mL P/S at 37°C in air.

### SDS-PAGE and Immunoblotting

At specified intervals after treatment, NIH-3T3 cells were washed twice in ice-cold PBS and scraped using ice-cold RIPA lysis buffer (50 mM Tris-HCl, pH 7.5; 150 mM NaCl; 1 mM EDTA; 0.5% sodium deoxycholate; 1% Triton X-100 and 0.1% SDS) or immunoprecipitation lysis buffer (10 mM Tris-HCl, pH 8.0; 150 mM NaCl; 2 mM EDTA; 10% glycerol and 1% NP40). Lysis buffer was freshly supplemented with 1X EDTA-free Complete protease (#05892791001, Roche) and 1X PhosStop phosphatase inhibitor cocktail (#04906845001, Roche). Extracted proteins (30 μg) were separated in 12% SDS polyacrylamide gels and then transferred onto nitrocellulose membranes (Amersham Bioscience). Protein extracts from 10 whole *Xenopus laevis* embryos were prepared as described [34] and loaded into a 12% SDS polyacrylamide gel. Membranes were blocked using TBS with 0.05% Tween20 (TBS-T) containing 5% non-fat dry milk (Biorad) or in 2% BSA for phospho-antibodies. The antibodies were anti-ERK2 (polyclonal rabbit IgG (C-14) and monoclonal mouse IgG2b (D-2) from Santa Cruz Biotechnology, 1:1000), anti-actin (polyclonal goat IgG (I-19) from Santa Cruz Biotechnology, 1:1000) and anti-GFP (monoclonal mouse IgG1κ, clones 7.1 and 13.1, from Roche (#11814460001), 1:1000). The phosphorylated forms of MAPK/ERK1/2 were detected using the anti-MAPK activated (diphosphorylated ERK1/2) antibody (monoclonal mouse IgG1, clone MAPK-YT, from Sigma Aldrich (M9692), 1:2000). HRP-conjugated secondary antibodies were anti-rabbit IgG, anti-mouse IgG or anti-goat IgG (whole antibody from Santa Cruz Biotechnology (sc-2004, sc-2005, sc-2020), 1:10000). Membranes were developed using the Luminata Classico Western HRP Chemiluminescence Detection Reagents (WBLUC0500, Millipore).

### Immunoprecipitation of MAPK/ERK2 and MBP phosphorylation assay

NIH-3T3 cell lysates (300 μg of protein) were immunoprecipitated directly as described [35]. Briefly, 50 μL of protein G magnetic beads (Millipore) per condition were washed and then conjugated with 1 μg of anti-ERK2 (C-14) or anti-GFP antibodies on a rotating wheel at 4°C overnight. Note that this anti-ERK2 antibody can also detect ERK1 although to a lesser extent. Antibodies against HA (#11583816001, Roche) were used as a control. The next day, antibody-conjugated beads were added to each sample and incubated on a rotating wheel at 4°C for 2 h. Supernatant was removed and beads were washed in lysis buffer. The MBP phosphorylation assay was performed on immunoprecipitated endogenous ERK2 and eGFP-ERK2 according to the manufacturer’s instructions (#2430444, Millipore). MBP proteins were subjected to SDS-PAGE (15% gel) and immunoblotted using an anti-phospho MBP antibody (monoclonal mouse IgG, clone p12, from Millipore (#05429), 1:1000).

### Immunofluorescence

NIH-3T3 cells were seeded in eight-well dishes (#80826, ibiTreat, Ibidi). At specified time intervals, cells were fixed in 4% paraformaldehyde in PBS for 10 min. Afterwards, cells were rinsed three times with PBS and permeabilized with 0.5% Triton X-100 in PBS for 5 min. Cells were then blocked in 2% FBS; 5% normal goat serum and 2% BSA in PBS) for 1 h at room temperature and with primary antibodies in blocking solution at 4°C overnight. The antibodies were anti-MAPK activated (diphosphorylated ERK1/2, 1:500), anti-ERK2 (D-2, 1:200) and anti-ERK1/2 (polyclonal rabbit IgG from Abcam (ab17942), 1:200). The next day, cells were rinsed three times with PBS and incubated in blocking solution containing anti-mouse Alexa Fluor 488 (polyclonal goat IgG from Life Technologies (A-10667), 1:500) and/or anti-rabbit Alexa Fluor 594 secondary antibodies (polyclonal goat IgG from Life Technologies (A-11012), 1:500) for 1 h at room temperature in the dark. After three more washes with PBS, slides were mounted in ProLong Gold anti-fading reagent (P36930, Life Technologies) and stored at 4°C in the dark.

### *Xenopus* embryo manipulation, RNA microinjection and immunostaining

Hormonal stimulation of female frogs, eggs collection, fertilization and dejellying of embryos were performed as previously described [36]. *Xenopus* embryos were staged as described [37]. Plasmids encoding eGFP-xERK2, 2A-eGFP-xERK2 and xMEK1-2A-eGFP-xERK2 were linearized with *Not*I, purified and transcribed using SP6 RNA polymerase and the mMessage mMachine kit (AM1340, Ambion, Life Technologies) according to the manufacturer’s instructions. Synthetic RNAs were purified with Chroma Spin column (#636073, Clontech). Embryos at the one-cell stage were microinjected with 500 pg of RNA. Embryos at different stages were collected for western blotting and immunostaining or directly processed for whole-embryo confocal microscopy. Blastula, gastrula and tailbud stage embryos were placed in an imaging chamber containing a layer of 2% agarose MP (#11388983001, Roche) and 1/10 Marc’s Modified Ringers (MMR) solution (1 M NaCl; 20 mM KCl; 10 mM MgCl_2_; 20 mM CaCl_2_ and 50 mM Hepes, pH 7.5). *Xenopus laevis* embryos were fixed in 4% paraformaldehyde at 4°C overnight with gentle shaking in glass vials and then processed for whole-mount immunostaining [38]. Antibodies were anti-ERK2 (D-2, 1:50) and Alexa Fluor 488 goat anti-mouse IgG secondary antibodies (1:100). Embryos were cut in half at the equator, and the animal half was mounted on a curved slide in ProLong Gold anti-fading reagent and stored at 4°C in the dark.

### Fluorescence imaging and data analysis

**Fig 1:**
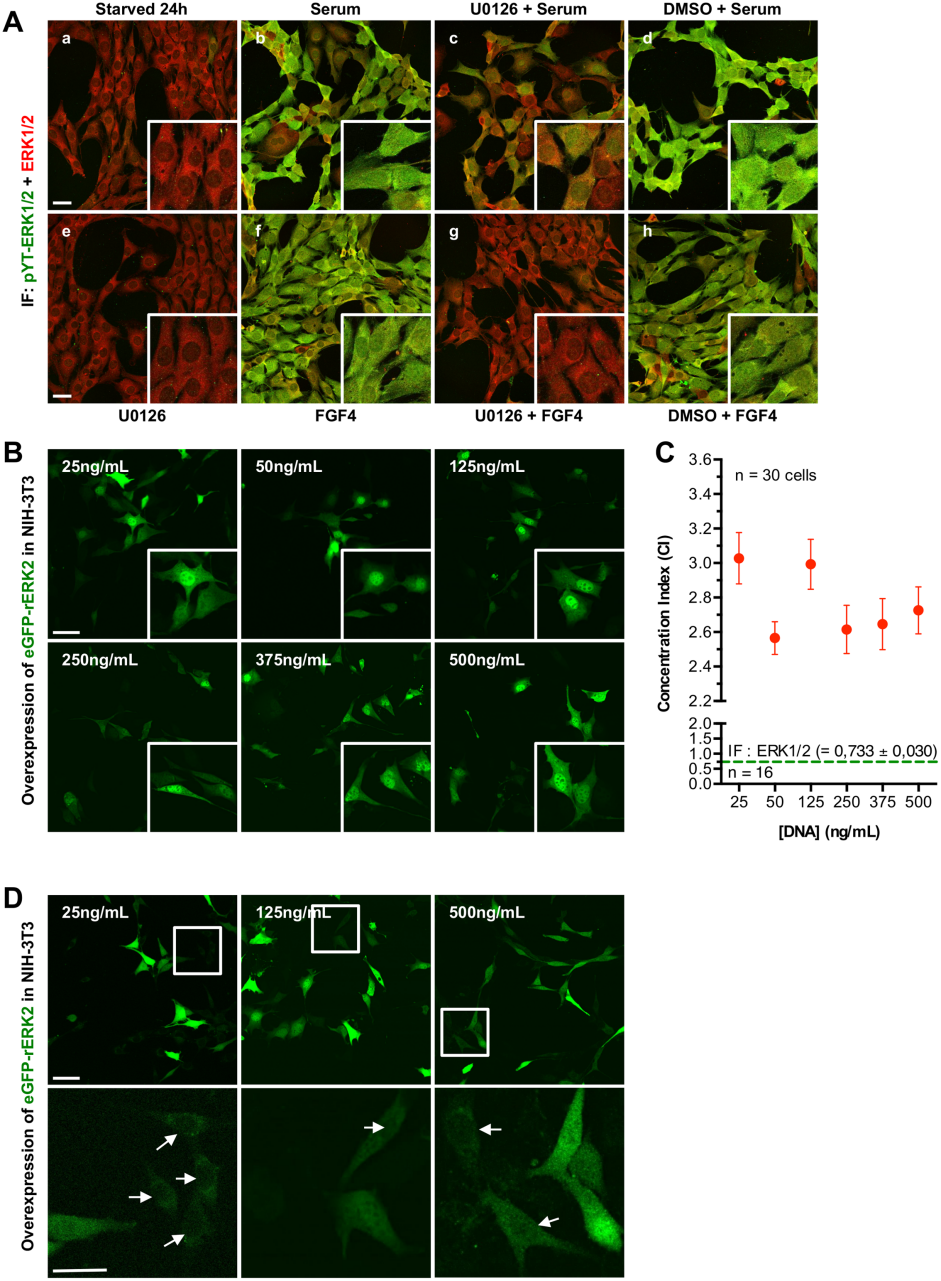
Overexpression of eGFP-rERK2 induces nuclear accumulation of eGFP-rERK2. **(A)** NIH3T3 cells were serum-starved for 24 h (a) and then stimulated with 10% serum (b) or 100 ng/mL FGF4 (f). In other conditions, cells were pretreated with 20 μM U0126 (c, e, g) or vehicle DMSO (d, h) for 30 min before stimulation with 10% serum (c, d) or FGF4 (g, h). Cells were fixed, processed for double immunofluorescence with antibodies against total ERK1/2 and activated di-phosphorylated YT-ERK1/2, and then imaged by confocal microscopy. A maximum-intensity projection of a 5-μm thick z-stack (step size: 0.3 μm) for each overlapping image is shown. **(B)** NIH-3T3 cells were transiently transfected with increasing amounts of eGFP-rERK2 plasmid as indicated on the top left of each image, serum-starved for 24 h, fixed, and then imaged by confocal microscopy. The total amount of DNA was kept at 500 ng/mL of medium in all conditions. Higher magnification images of representative eGFP-rERK2 localization are shown in white squares (bottom right). Scale bars: 50 μm. **(C)** Relationship between the concentration of eGFP-rERK2 plasmid and the concentration index (CI). Higher CI values reflect greater accumulation of eGFP-ERK2 in the nucleus. Average CI was determined by examination of at least 30 randomly selected cells for each of the transfected conditions from two independent experiments. Average CI value for endogenous ERK1/2 in serum-starved NIH-3T3 is also shown (green dotted line). **(D)** NIH-3T3 cells transfected with 25, 125 or 500 ng/mL of eGFP-rERK2 were observed under severe imaging conditions to visualize cells that express very low level of eGFP-rERK2 protein (upper panel, white squares). Higher magnification images of these cells exhibiting mainly cytoplasmic localization of eGFP-rERK2 are also shown (bottom panel, white arrows). Scale bars: 50 μm.

Live-cell, immunofluorescence imaging and FRAP experiments were all performed with an inverted confocal Leica TCS SP5 X microscope (DMI6000, Leica Microsystems). For all experiments, a 63x/1.2NA water immersion objective was used, except for the result shown in Fig 1A (40x/1.3NA oil immersion objective). Image size was 1024 x 1024 pixels and the zoom factor was 1, for a pixel size of 0.5 μm. The confocal pinhole was set to 1.0 Airy, for a 0.921 μm optical slice. Laser sources were either a white light laser (Koheras) for live-cell imaging and immunofluorescence preparations, or an argon laser (Leica Microsystems) used at a wavelength of 488 nm for FRAP experiments. All experiments were performed at 37°C.

To quantify ERK2 nuclear translocation, we measured the average fluorescence intensity in the nucleus (*Fnuc*) and in the cytoplasm (*Fcyto*) to determine a nucleo-cytoplasmic concentration index (CI) calculated as follows:

$$CI=\frac{Fnuc-BG}{Fcyto-BG}$$

where *BG* corresponds to the average fluorescence intensity background. Fluorescence intensity in fixed cells was quantified manually using ImageJ software (National Institutes of Health) by drawing specific ROIs in the nucleus and cytoplasm, and outside the cell for background. Fluorescence intensity quantification of ERK2-LOC dynamics in single cells was done automatically with Volocity image analysis software (Perkin Elmer), which required segmentation of fluorescence labeled cells throughout the entire time-lapse. This was done by incubating the cells with Hoechst before starting the experiment to discriminate nuclei from whole cells and to define a mask around each nucleus. Cytoplasm fluorescence intensity of ERK2-LOC was then measured by subtracting fluorescence intensity due to the nucleus from that of the whole cell.

Whole *Xenopus laevis* embryos at early stages of development were imaged with an upright confocal Nikon A1 microscope (Eclipse FN1, Nikon). A 25x/1.1NA objective lens was used, and pinhole was set to 0.6 Airy for an optical slice of 1.04 μm. Image size was 1024 x 1024 pixels acquired at a speed of 0.5 frames/sec (scanning speed of 512 Hz). The zoom factor was 1 for a pixel size of 0.5 μm. 3D reconstructions of the confocal z-stack images were performed using NIS Elements 4.3 software (Nikon) and ImageJ (National Institutes of Health). *Xenopus laevis* embryos tailbud stage were imaged on an upright Nikon Eclipse 80i epifluorescence microscope equipped with a 4x/0.13NA Plan Fluor objective (Nikon) and a CoolSNAP ES CCD Photometrics camera (Roper Scientific).

### Fast-FRAP experiments

In Fast-FRAP experiments, pre-bleach acquisition, bleaching and fluorescence recovery measurements were performed by repeatedly scanning one line across a targeted cell. Scanning was bidirectional at 1400 Hz. The zoom factor was 5, yielding a pixel size of 0.048 μm. Resulting X(t) images were 1024 x 14416 pixels. Pre-bleach acquisition was carried out to compensate for the loss of fluorescence due to the acquisition. A bleaching ROI was set across either the cell nucleus or the cytoplasm. Fast-FRAP acquisition was as follows: 1 sec of pre-bleach acquisition, 150 ms of bleaching, and 3 sec of fluorescence recovery measurements. Bleaching was achieved with the laser operating at 95% power with the AOTF set to 100%. For imaging, laser power was attenuated to 2% of the AOTF. Fluorescence was detected between 500 nm and 570 nm. Fluorescence recovery curves were exported and analyzed using LAS AF and MATLAB (MathWorks). Curve normalization was done using the “double normalization” formula [39]:

$$I_{FRAP\;NORM}=\;\frac{I_{Ref\;Pre}}{I_{Ref}\left(t\right)}\;\cdot \frac{I_{FRAP}\left(t\right)}{I_{FRAP\;Pre}}$$

with *I*_*FRAP*_(*t*) as the fluorescense intensity in the FRAP ROI, *I*_*Ref*_(*t*) as the reference fluorescence intensity along the same line scan, *I*_*FRAP Pre*_ as the mean fluorescence intensity before bleaching in the FRAP ROI, and *I*_*Ref Pre*_ as the reference for mean fluorescence intensity before bleaching along the same line scan. All measurements were corrected for background noise.

### Statistics

Results are presented as means ± SEM. Statistical analyses were performed using PRISM 6.0 software (GraphPad). One-way and two-way ANOVA and Dunnett’s test, accepting *p* ≤ 0.05 as significant, as well as a two-tailed unpaired *t*-test were used to compare CI values. Curves of FRAP experiments were fitted by one-phase exponential equations. Differences between two groups for half-life recovery and percentage of immobile fraction were analyzed using a two-tailed unpaired *t*-test. The cleavage efficiency of T2A peptide, the expression level of tagged-ERK2 and the ratio of phospho/total ERK2 were quantified by densitometry using Image J (National Institutes of Health).

## Results

### Validation of endogenous ERK1/2 dynamics in NIH3T3 cells

To ensure that our cellular system behaves as described [21], the phosphorylation profile and subcellular localization of endogenous ERK1/2 in NIH-3T3 fibroblast cells were examined using specific activators and/or inhibitors of the pathway (Fig 1A). In non-stimulated conditions, ERK1/2 displayed a basal phosphorylation and was localized mainly in the cytoplasm (a). Pretreatment of non-stimulated cells with U0126 reduced this phosphorylation (e). Phosphorylation of ERK1/2 induced by serum (10%) or FGF4 (100 ng/mL) resulted in ERK1/2 nuclear translocation and homogenous distribution of ERK1/2 throughout the cells (b and f). U0126 pretreatment dramatically decreased both serum- and FGF4- induced phosphorylation of ERK1/2 and prevented the nuclear accumulation of ERK1/2 (c and g). However, ERK1/2 phosphorylation was not completely abolished when cells were simultaneously treated with U0126 and serum: phosphorylation signals were still detectable in the cytoplasm and the nucleus (c). In contrast, neither phosphorylation nor localization of ERK1/2 was altered when cells were treated with DMSO and either serum or FGF4 (d and h). These results are in accordance with previous reports [21] and demonstrate the proper functioning of our biological system.

### Artefacts in localization of over-expressed eGFP-rEKR2 in NIH-3T3 cells

**Fig 2:**
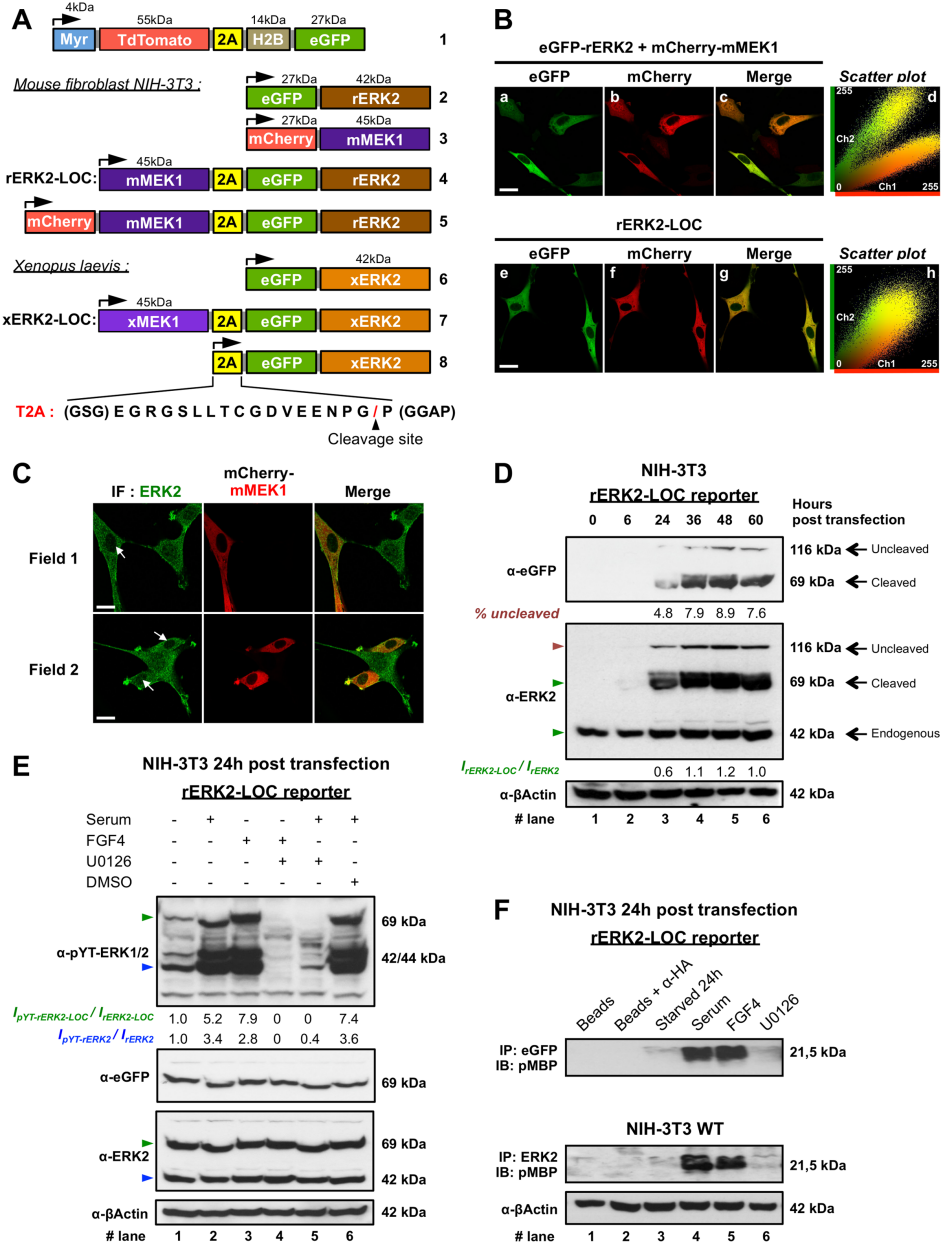
Equimolar co-expression of eGFP-rERK2 and mMEK1 restores cytoplasmic localization of eGFP-rERK2. **(A)** Schematic representation of all genetically encoded molecular constructs used in this study. The corresponding amino acid sequence of 2A (yellow box) encodes a T2A peptide isolated from plasmid Myr-TdTomato-2A-H2B-eGFP (#1). Amino acids (GSG) and (GGAP) improve cleavage efficiency. The red slash symbol at the peptide C-terminal end indicates the 2A peptide cleavage site. **(B)** Fluorescence confocal imaging of NIH-3T3 cells after transfection with different plasmids and serum starvation for 24 h: top, transfection with eGFP-rERK2 (#2) and mCherry-mMEK1 (#3) plasmids; bottom, transfection with mCherry-mMEK1-2A-eGFP-rERK2 (#5). Representative images are shown of rERK2 protein distribution (green: a, e), mMEK1 distribution (red: b, f) and the merged image (c, g). Corresponding scatter plots of green and red intensities of each pixel on the whole images are shown (d, h). Co-localized pixels are visualized in yellow. Scale bar: 20 μm. **(C)** Fluorescence confocal imaging of NIH-3T3 cells transfected with mCherry-mMEK1 (middle, red) and labeled with an anti-ERK2 antibody (left, green) after 24 h of serum starvation. Co-localization of rERK2 and mCherry-mMEK1 is shown in the merged images (right, yellow). White arrows point to nuclei of transfected cells. Scale bar: 20 μm. **(D)** Western blot analysis of NIH-3T3 cells transfected with rERK2-LOC at the indicated time-points. Cell lysates were analyzed by immunoblotting with the indicated antibodies (left of each blot). The percentage of uncleaved polypeptide (full-length mMEK1-2A-GFP-rERK2, red triangle) was quantified by densitometry. Quantitative comparison of the levels of overexpressed rERK2-LOC and endogenous ERK2 (green triangles, middle panel) is indicated below the blot as ***I***_***rERK2-LOC***_ ***/ I***_***rERK2***_. **(E)** After 24 h of serum starvation, NIH-3T3 cells transfected with rERK2-LOC were left untreated or were pretreated for 1 h with U0126 or DMSO, and then stimulated with serum or FGF4 for 15 min. Corresponding cell lysates were immunoblotted with the indicated antibodies (left of each blot). Relative phosphorylation levels of rERK2-LOC (green triangles) and endogenous ERK2 (blue triangles) were measured by densitometry. The ratios of phosphorylated protein to total proteins (***I***_***pYT-rERK2-LOC***_ ***/ I***_***rERK2-LOC***_ and ***I***_***pYT-ERK2***_ ***/ I***_***ERK2***_) are indicated below the top blot. **(F)** rERK2-LOC—transfected NIH-3T3 cells were serum starved for 24 h and then left untreated or incubated or not with U0126 for 1 h before stimulation with serum or FGF4 for 15 min. Cells lysates were immunoprecipitated with anti-eGFP (top panel) or anti-ERK2 antibodies (middle panel), and ERK1/2 kinase activity was assayed *in vitro*. The phosphorylated form of MBP (pMBP) was detected by immunoblotting. Unconjugated beads and beads conjugated with anti-HA antibodies were used as a control in the assays. Lysate inputs for immunoprecipitation were probed with anti-β-actin antibody as a loading control. At least two independent experiments and 15 cells were measured from fixed cells. Biochemical data are representative of at least two independent experiments.

We assessed the subcellular distribution of overexpressed eGFP-ERK2 in NIH-3T3 cells in serum-starved and non-stimulated conditions. We documented ERK2 localization in NIH-3T3 cells transiently transfected with the rat ERK2 (rERK2) fused to the C-terminus of the enhanced green fluorescent protein (eGFP-rERK2). Note that the eGFP-rERK2 function was not altered as previously reported [29]. To obtain various eGFP-rERK2 expression levels within a population of NIH-3T3 cells, they were transiently transfected with eGFP-rERK2 plasmids in concentrations ranging from 25 to 1000 ng/mL to generate dim and bright fluorescence. The subcellular distribution of overexpressed eGFP-ERK2 in serum-starved and non-stimulated NIH-3T3 cells is presented in Fig 1B. eGFP-rERK2 accumulated in the nucleus of brightly fluorescent cells but was homogenously distributed between cytoplasm and nucleus in weakly fluorescent cells. This pattern was confirmed by the nucleo-cytoplasmic concentration index (CI) results (Fig 1C, red dots), which are different from those of endogenous ERK1/2 (CI = 0.733 ± 0.030, n = 16,) in similar experimental conditions (Fig 1Aa and 1C, green dotted line). The differences observed in CI values among plasmid concentration (Fig 1C) reflect important cell-to-cell variations in eGFP-rERK2 expression and thus in eGFP-rERK2 subcellular distribution. eGFP-ERK2 in serum-starved NIH-3T3 cells across the range of plasmid concentrations used was faithfully detected only in the weakly fluorescent cells (Fig 1D) and when the previously described severe imaging conditions were used [29]. Consistent with previous studies, plasmids encoding eGFP-rERK2 and mCherry-mMEK1 (Fig 2A, #2 and #3) were transiently co-transfected in equal amounts (final concentration of 1 μg/mL) to avoid saturation of ERK2 binding partners in the cytoplasm and subsequent nuclear accumulation of eGFP-rERK2 [26,27]. Twenty-four hours after transfection and serum starvation, confocal images of fluorescent single cells in the red and green channels showed a marked heterogeneous expression of mCherry-mMEK1 and eGFP-rERK2 due to transient transfection (Fig 2B, upper panel). In cells coexpressing eGFP-rERK2 and mCherry-mMEK1 in similar proportions, eGFP-rERK2 was localized in the cytoplasm. But cells expressing more eGFP-rERK2 in comparison to mCherry-mMEK1 showed a more prominent nuclear localization of the kinase (Fig 2Bc and 2Bd).

### Faithful eGFP-ERK2 localization restored in living NIH3T3 cells

To provide an accurate and faithful read-out of the subcellular distribution of ERK2 regardless of its expression level, and to monitor the spatiotemporal signature of ERK2 in living cells by fluorescence imaging, we constructed and validated a novel molecular tool: rERK2-LOC (Fig 2A, #4). Based on previous reports and our own observations (Figs 1 and 2Ba–2Bd), we reasoned that over-expression of eGFP-rERK2 should be counter-balanced by coexpression of equal amounts of mMEK1, the main interacting partner of ERK2, in order to maintain the system’s equilibrium. To that end, we used the T2A peptide, which functions as a reliable ribosomal skip mechanism to produce multiple polypeptides from a unique translation start site (Fig 2Aa and Materials and Methods).

To assess mouse ERK2 localization in serum-starved conditions, NIH-3T3 cells transiently transfected with the mCherry-mMEK1-2A-eGFP-rERK2 plasmid (Fig 2B, lower panel) were imaged by fluorescence confocal microscopy. eGFP-rERK2 and mCherry-mMEK1 were co-expressed in all transfected cells, as observed in the overlay image (Fig 2Bg). Co-localization analysis based on the generation of a scatter-plot on the whole image of red intensities *versus* green intensities for each pixel confirmed co-localization and indicated comparable expression levels of ERK2 and MEK1 (Fig 2Bh). The results show that T2A mediated the equimolar coexpression of eGFP-rERK2 and mCherry-mMEK1 and that cytoplasmic localization of eGFP-ERK2 was restored in serum-starved, non-stimulated cells regardless of the expression level (Fig 1Aa). However, the nuclei of transfected cells appear “darker” than the nuclei of non-transfected cells harboring a more uniform distribution of endogenous ERK1/2 between the cytoplasm and the nucleus (Figs 1Aa and 2C, ERK2 immunostaining). We hypothesized that this could be due to the disruption of the initial MEK1/ERK2 ratio in NIH-3T3 cells after T2A-mediated coexpression of eGFP-rERK2 and mCherry-mMEK1 [40]. To increase the proportion of MEK1 with respect to that of endogenous ERK2, NIH-3T3 cells were then transfected with mCherry-mMEK1, serum starved for 24 h, and then immunostained for total ERK2 (Fig 2C). Interestingly, mCherry-mMEK1 overexpression decreased the level of endogenous ERK2 in the nucleus, consistent with our previous observations (Fig 2Be).

### Functional validation of the 2A-mediated eGFP-rERK2 and rMEK1 coexpression system

Analysis of western blot data from NIH3T3 cells transfected with the mMEK1-2A-eGFP-rERK2 plasmid and harvested 0, 6, 24, 36, 48, and 60 h later showed that eGFP-rERK2 expression was detectable 24 h after transfection and remained stable from 36 h (Fig 2D, upper and middle panel). Interestingly, the average abundance of eGFP-rERK2 ranged from 0.6- to 1.2-fold relative to that of endogenous ERK2 (Fig 2D, below middle panel) normalized against actin (Fig 2D, lower panel). Average cleavage efficiency decreased slightly over time from 95.2% to a minimum of 92.1% (Fig 2D, below upper panel). All experiments were performed 24 h after transfection, when average cleavage efficiency and relative abundance were optimal. rERK2-LOC was validated by monitoring its phosphorylation status (Fig 2E) and kinase activity (Fig 2F) on NIH3T3 cells transfected with rERK2-LOC upon activation and/or inhibition of the ERK1/2 signaling pathway. Similar phosphorylation patterns were observed for rERK2-LOC and endogenous ERK1/2 (Fig 2E, upper panel). rERK2-LOC and endogenous ERK2 were substantially phosphorylated in response to serum and FGF4 compared to absence of stimulation. However, densitometry (below the upper panel) revealed a pronounced effect of serum (between 5.2- and 7.4-fold higher, lanes 2 & 6) and FGF4 (7.9-fold higher, lane 3) relative to the basal phosphorylation of rERK2-LOC (lane 1) when compared with the phosphorylation status of endogenous ERK2 (between 3.4- to 3.6-fold higher in serum-treated conditions (lanes 2 & 6) and 2.8-fold higher in FGF4-treated condition (lane 3) relative to the basal value. Pretreatment with U0126 prevented serum- and FGF4-induced phosphorylation of both rERK2-LOC and endogenous ERK2; no phosphorylation signal was detectable in lanes 4 and 5, except for endogenous ERK2 in lane 5, which has a 0.4-fold phosphorylation signal relative to the basal value. In a complementary approach, MBP-based *in vitro* kinase assay was used to determine the kinase activities of rERK2-LOC (Fig 2F, upper panel) and endogenous ERK2 (Fig 2F, middle panel). Phospho-MBP (p-MBP) immunoblotting showed that rERK2-LOC and endogenous ERK2 had equivalent phosphorylation capabilities in cells treated with serum or FGF4, demonstrating the functional kinase activity of rERK2-LOC. Treatment with U0126 impaired the kinase activity of both rERK2-LOC and endogenous ERK2 alike. The results of biochemical assays show that eGFP-rERK2 was coexpressed with mMEK1 by means of the 2A system, and that it fulfills the biochemical functions of endogenous ERK2 in NIH3T3 cells.

### Enhanced contrast monitoring of eGFP-ERK2 in living cells

**Fig 3:**
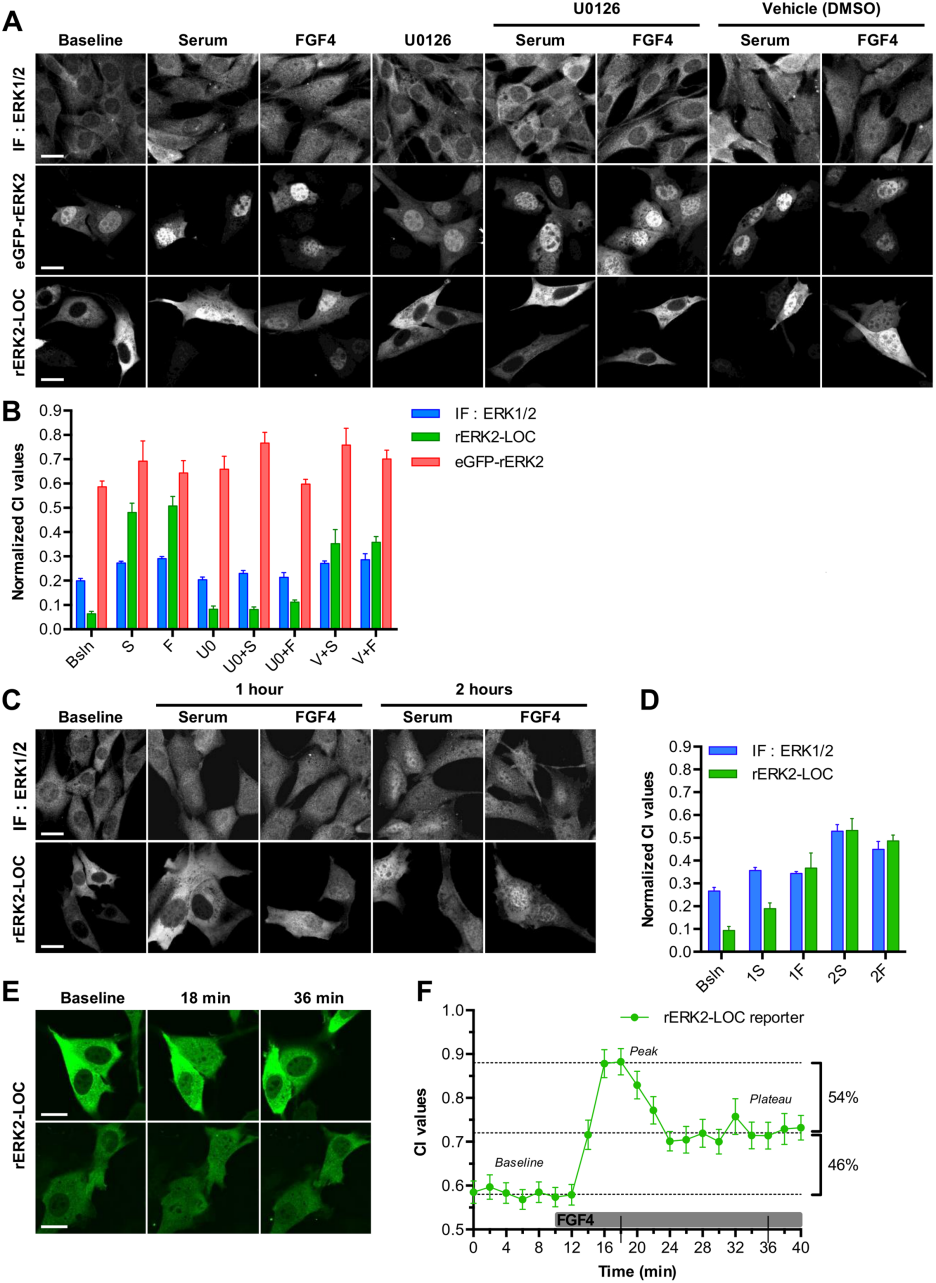
rERK2-LOC expression faithfully reports localization of ERK2. **(A)** Non-transfected (top row) and transfected NIH-3T3 cells overexpressing either eGFP-rERK2 (middle row) or rERK2-LOC (bottom row) were serum starved for 24 h, and then were left untreated or were treated with U0126 or DMSO for 1 h. Next, they were stimulated with serum or FGF4 for 15 min, or were left unstimulated (baseline). All cells were fixed and non-transfected cells were processed for immunofluorescence using the anti-ERK1/2 antibody (top row) and all cells were imaged by confocal microscopy. Shown are representative images of ERK2 localization under the different treatments. Scale bars: 20 μm. **(B)** Quantitative comparison of the nucleo-cytoplasmic concentration index (CI) of ERK2 between endogenous ERK1/2 (blue bars), overexpressed eGFP-rERK2 (red bars) and rERK2-LOC (green bars). CI values were normalized between 0 and 1 ($\bar{CI}$ values), where 0 and 1 are respectively the minimal and maximal CI values obtained. Bsln: baseline, S: serum, F: FGF4, U0: U0126, V: vehicle (DMSO). **(C)** Non-transfected (top row) and transfected NIH-3T3 cells overexpressing rERK2-LOC (bottom row) were serum starved for 24 h and then stimulated with serum or FGF4 for 1 or 2 h, or left untreated (baseline). All cells were fixed and non-transfected cells were processed for immunofluorescence using the anti-ERK1/2 antibody (top row) and all cells were imaged by confocal microscopy. Shown are representative images of ERK2 localization under the different treatments. Scale bar: 20 μm. **(D)** Quantitative comparison of nucleo-cytoplasmic concentration index (CI) of ERK2 at the indicated time-points between endogenous ERK1/2 (blue bars) and overexpressed rERK2-LOC (green bars). CI values were normalized between 0 (minimum obtained) and 1 (maximum obtained) ($\bar{CI}$ values). Bsln: baseline; 1S and 1F: 1 h serum and 1 h FGF4; 2S and 2F: 2 h serum and 2 h FGF4. **(E)** Monitoring of the subcellular distribution of rERK2-LOC in (24h) serum-starved NIH-3T3 cells by time-lapse confocal microscopy every 2 min for 10 min (baseline) and after FGF4 stimulation (100 ng/mL) for 30 min. **(F)** Nuclear and cytoplasmic intensities of each rERK2-LOC transfected cell were measured with Volocity software for each time-point to calculate the concentration index values (CI). Vertical error bars represent the average ± SEM. Two-way ANOVA test, accepting *p* ≤ 0.05 as significant, was performed to compare $\bar{CI}$ values differences between endogenous ERK1/2, eGFP-rERK2 and rERK2-LOC for a same treatment. One-way ANOVA test, accepting *p* ≤ 0.05 as significant, was performed to compare $\bar{CI}$ values among all the treatments (Tables 2 and 3). At least two independent experiments were performed. The number of cells per condition (n) from fixed cells is indicated in Tables 2 and 3 for statistical analysis; at least 80 cells were measured for time-lapse microscopy.

The relevance and the faithfulness of our novel ERK2 localization reporter were further characterized. The subcellular distributions of overexpressed eGFP-rERK2 and rERK2-LOC were examined and compared to immuno-localized endogenous ERK1/2 proteins monitored by fluorescence imaging on fixed NIH-3T3 cells following different treatments. In accordance with our other observations (Fig 1B), overexpressed eGFP-rERK2 accumulated heavily in the nucleus in both non-stimulated and treated cells regardless of the treatment (Fig 3A, middle panel). By contrast, T2A-mediated MEK1/ERK2 coexpression resulted in a subcellular distribution of rERK2-LOC (Fig 3A, lower panel) like that of endogenous ERK1/2 (Fig 3A, upper panel). The results show that rERK2-LOC localization was strictly cytoplasmic when the ERK1/2 signaling pathway was inhibited, and that it accumulated in the nucleus when the pathway was activated.

To quantify the ERK2 subcellular distribution in different experimental conditions, CI values were normalized between 0 and 1 (abbreviated $\bar{CI}$ values) (Fig 3B) and are listed in Table 2. We noticed no significant variations in overexpressed eGFP-rERK2 subcellular distribution regardless of the treatment used to activate or inhibit the ERK1/2 pathway, except for $\bar{CI}$_U0+S_ and $\bar{CI}$_V+S_ (Table 2). More importantly, incubation of the cells with U0126 in the presence or absence of serum or FGF4 failed to reestablish the cytoplasmic localization of eGFP-rERK2. Normalized CI values for eGFP-rERK2 transfected cells treated with serum ($\bar{CI}$_S_) or U0126 and serum ($\bar{CI}$_U0+S_) were respectively 1.1- and 1.3-fold higher than non-stimulated cells (referred as baseline condition, $\bar{CI}$_Bsln_) (0,692 ± 0,083, n = 5 and 0.767 ± 0.043, n = 6 versus 0.586 ± 0.024, n = 10; *p* ≤ 0.05), indicating that eGFP-ERK2 concentrated much more in the nucleus in U0126 condition. These data do not agree with the effect of treatments on endogenous ERK1/2 (Table 2). Remarkably, consistent with the data in Fig 2B and 2C, $\bar{CI}$_Bsln_ as well as $\bar{CI}$_U0_ values for cells expressing rERK2-LOC were respectively 3.1- and 2.5-fold lower than that of endogenous ERK1/2 in non-stimulated conditions (0.064 ± 0.009, n = 12 versus 0.200 ± 0.009, n = 16; *p* ≤ 0.001) (Table 2). Whereas in non-stimulated cells rERK2-LOC was localized mainly in the cytoplasm, stimulation by serum or FGF4 provoked nuclear accumulation that was markedly enhanced relative to endogenous ERK1/2. $\bar{CI}$ values of rERK2-LOC in cells stimulated with serum or FGF4 were 7.5- and 7.9-fold higher than non-stimulated cells, respectively. In comparison, $\bar{CI}$ values of endogenous ERK1/2 were 1.4- and 1.5-fold higher than baseline value under the same experimental conditions (Table 2). The difference of averaged $\bar{CI}$ values (Δ$\bar{CI}$) between baseline and serum or FGF4 stimulation for rERK2-LOC was 5.7- and 4.9-fold higher than that for endogenous ERK1/2, confirming several orders of magnitude in the nuclear translocation for rERK2-LOC (Table 2). In addition, treatment with U0126 alone or combined with serum or FGF4 caused no significant change in $\bar{CI}$ for rERK2-LOC, in accordance with endogenous ERK1/2 (Table 2).

**Table 2:** Statistical analysis of $\bar{CI}$ and Δ$\bar{CI}$ values for overexpressed eGFP-ERK2, endogenous ERK1/2 and rERK2-LOC, 15 min after serum or FGF4 stimulation.

	Treatments	$\bar{CI}$	n	S	*p* value	Fold-change	Δ$\bar{CI}$	Ratio rERK2-LOC / IF:ERK1/2
**eGFP-rERK2**	Baseline	0.586 ± 0.024	10					
	Serum	0.692 ± 0.083	5	ns	0.4658	1.18		
	FGF4	0.643 ± 0.050	6	ns	0.9065	1.10		
	U0126	0.659 ± 0.054	8	ns	0.6977	1.13		
	U0126 + serum	0.767 ± 0.043	6	*	0.0355	1.31		
	U0126 + FGF4	0.598 ± 0.019	11	ns	0.9997	1.02		
	DMSO + serum	0.759 ± 0.068	7	*	0.0353	1.30		
	DMSO + FGF4	0.701 ± 0.036	10	ns	0.1933	1.20		
**IF:ERK1/2**	Baseline	0.200 ± 0.009	16					
	Serum	0.273 ± 0.007	9	***	0.0003	1.37	0.073 ± 0.011	
	FGF4	0.291 ± 0.008	27	****	< 0.0001	1.46	0.091 ± 0.012	
	U0126	0.204 ± 0.011	13	ns	0.9996	1.02	0.004 ± 0.014	
	U0126 + serum	0.230 ± 0.012	12	ns	0.2652	1.15	0.030 ± 0.016	
	U0126 + FGF4	0.214 ± 0.019	7	ns	0.957	1.07	0.014 ± 0.021	
	DMSO + serum	0.271 ± 0.010	18	****	< 0.0001	1.36	0.071 ± 0.013	
	DMSO + FGF4	0.287 ± 0.025	6	***	0.0002	1.44	0.087 ± 0.026	
**rERK2-LOC**	Baseline	0.064 ± 0.009	12					
	serum	0.480 ± 0.007	7	****	< 0.0001	7.50	0.416 ± 0.041	5.70
	FGF4	0.508 ± 0.008	16	****	< 0.0001	7.94	0.444 ± 0.040	4.88
	U0126	0.083 ± 0.011	15	ns	0.9952	1.30	0.019 ± 0.016	4.75
	U0126 + serum	0.082 ± 0.012	6	ns	0.9994	1.28	0.018 ± 0.014	0.60
	U0126 + FGF4	0.112 ± 0.019	11	ns	0.7481	1.75	0.048 ± 0.013	3.43
	DMSO + serum	0.353 ± 0.010	7	****	< 0.0001	5.52	0.289 ± 0.058	4.07
	DMSO + FGF4	0.358 ± 0.025	18	****	< 0.0001	5.60	0.294 ± 0.026	3.38

Statistical significance for differences among overexpressed eGFP-rERK2, endogenous ERK1/2 and rERK2-LOC was tested by one-way ANOVA and Dunnett’s test, accepting *p* ≤ 0.05 as significant. The ratio between Δ$\bar{CI}$ *o*f endogenous ERK1/2 and rERK2-LOC shows the differences in magnitude order in function of the treatment. Symbols: $\bar{CI}$, average of CI values ± SEM; Δ$\bar{CI}$, difference between means compared to baseline value as reference; SEM, Standard Error of Mean; n, number of cells analyzed; S: statistically significant; ns, p > 0.05;

*, p ≤ 0.05;

**, p ≤ 0.01;

***, p ≤ 0.001;

****, p ≤ 0.0001.

As several studies reported that simple coexpression of MEK1 with tagged-ERK2 derived from different plasmids disturbed the distribution of tagged ERK2, evidenced by its abnormally short persistence in the nucleus upon stimulation [26,27,30], we monitored the localization of rERK2-LOC and compared it with that of endogenous ERK1/2 at 1 and 2 h after serum or FGF4 stimulation (Fig 3C). Consistent with a previous study [21], endogenous ERK1/2 was distributed relatively homogenously throughout the cells 1 h after addition of serum or FGF4 (Fig 3C, upper panel). But 2 h after serum or FGF4 stimulation, it accumulated in the nucleus. Surprisingly, the results clearly show that rERK2-LOC mimicked endogenous ERK1/2 in response to the different treatments (Fig 3C, bottom panel) and exhibited progressive nuclear accumulation in serum-starved NIH-3T3 cells treated with serum or FGF4 (Fig 3D). $\bar{CI}$ values of rERK2-LOC after 2 h in serum- or FGF4-stimulated conditions were 5.7- and 5.2-fold higher than baseline, respectively. In comparison, $\bar{CI}$ values of endogenous ERK1/2 were only 2.0- and 1.7-fold higher than baseline under the same experimental conditions (Table 3). The difference of averaged $\bar{CI}$ values (Δ$\bar{CI}$) between baseline and 2 h serum or 2 h FGF4 stimulation for rERK2-LOC was 1.7- and 2.2-fold higher than that for endogenous ERK1/2, confirming that late nuclear accumulation is also markedly enhanced using rERK2-LOC (Table 3).

**Table 3:** Statistical analysis of $\bar{CI}$ and Δ$\bar{CI}$ values for endogenous ERK1/2 and rERK2-LOC, 1 h and 2 h after serum or FGF4 stimulation.

	Treatments	$\bar{CI}$	n	S	*p* value	Fold-change	Δ$\bar{CI}$	Ratio rERK2-LOC / IF:ERK1/2
**IF:ERK1/2**	Baseline	0.267 ± 0.016	18					
	1 h—serum	0.357 ± 0.013	21	**	0.0066	1.34	0.090 ± 0.021	
	1 h—FGF4	0.343 ± 0.009	27	*	0.0172	1.29	0.077 ± 0.018	
	2 h—serum	0.529 ± 0.029	19	****	< 0.0001	1.98	0.262 ± 0.033	
	2 h—FGF4	0.449 ± 0.035	14	****	< 0.0001	1.68	0.183 ± 0.038	
**rERK2-LOC**	Baseline	0.093 ± 0.018	13					
	1 h—serum	0.189 ± 0.025	11	ns	0.4524	2.03	0.096 ± 0.031	1.07
	1 h—FGF4	0.367 ± 0.066	15	***	0.0003	3.95	0.274 ± 0.069	3.56
	2 h—serum	0.532 ± 0.053	16	****	< 0.0001	5.72	0.439 ± 0.055	1.68
	2 h—FGF4	0.486 ± 0.026	12	****	< 0.0001	5.23	0.393 ± 0.032	2.15

Statistical significance for differences among endogenous ERK1/2 and rERK2-LOC was tested by one-way ANOVA and Dunnett’s test, accepting *p* ≤ 0.05 as significant. The ratio between Δ$\bar{CI}$ of endogenous ERK1/2 and rERK2-LOC shows the differences in magnitude orders in function of the treatment. Symbols: $\bar{CI}$, average of CI values ± SEM; Δ$\bar{CI}$, difference between means compared to baseline value as reference; SEM, Standard Error of Mean; n, number of cells analyzed; S: statistically significant; ns, p > 0.05;

*, *p* ≤ 0.05;

**, *p* ≤ 0.01;

***, *p* ≤ 0.001;

****, *p* ≤ 0.0001.

To monitor rERK2-LOC dynamics at higher temporal resolution in living NIH-3T3 cells, we used automated time-lapse confocal microscopy with a temporal resolution of 2 min for 40 min. After a baseline period of 10 min (CI = 0.582 ± 0.004), rERK2-LOC entered the nucleus between 2 and 4 min after FGF4 addition and reached a maximum within 8 min after stimulation (CI = 0.880 ± 0.002) (Fig 3E and 3F and S1 Movie). These results are in agreement with previous studies on ERK2 translocation kinetics following FGF4 treatment on NIH-3T3 cells [29]. Whereas a sustained ERK2 nuclear localization was reported in similar experimental conditions [21,29], our results show a decrease of 54% from the initial peak, but sill 46% above the baseline for the remaining time of the experiment (CI = 0.719 ± 0.006). Taken together, these results show that our novel molecular reporter of ERK2 localization substantially set up the monitoring of ERK2. It provides an emphasized relocation of the coexpressed ERK2 while remaining faithful to that of the endogenous under all experimental conditions.

### rERK2-LOC provides a relevant read-out for ERK2 mobility

**Fig 4:**
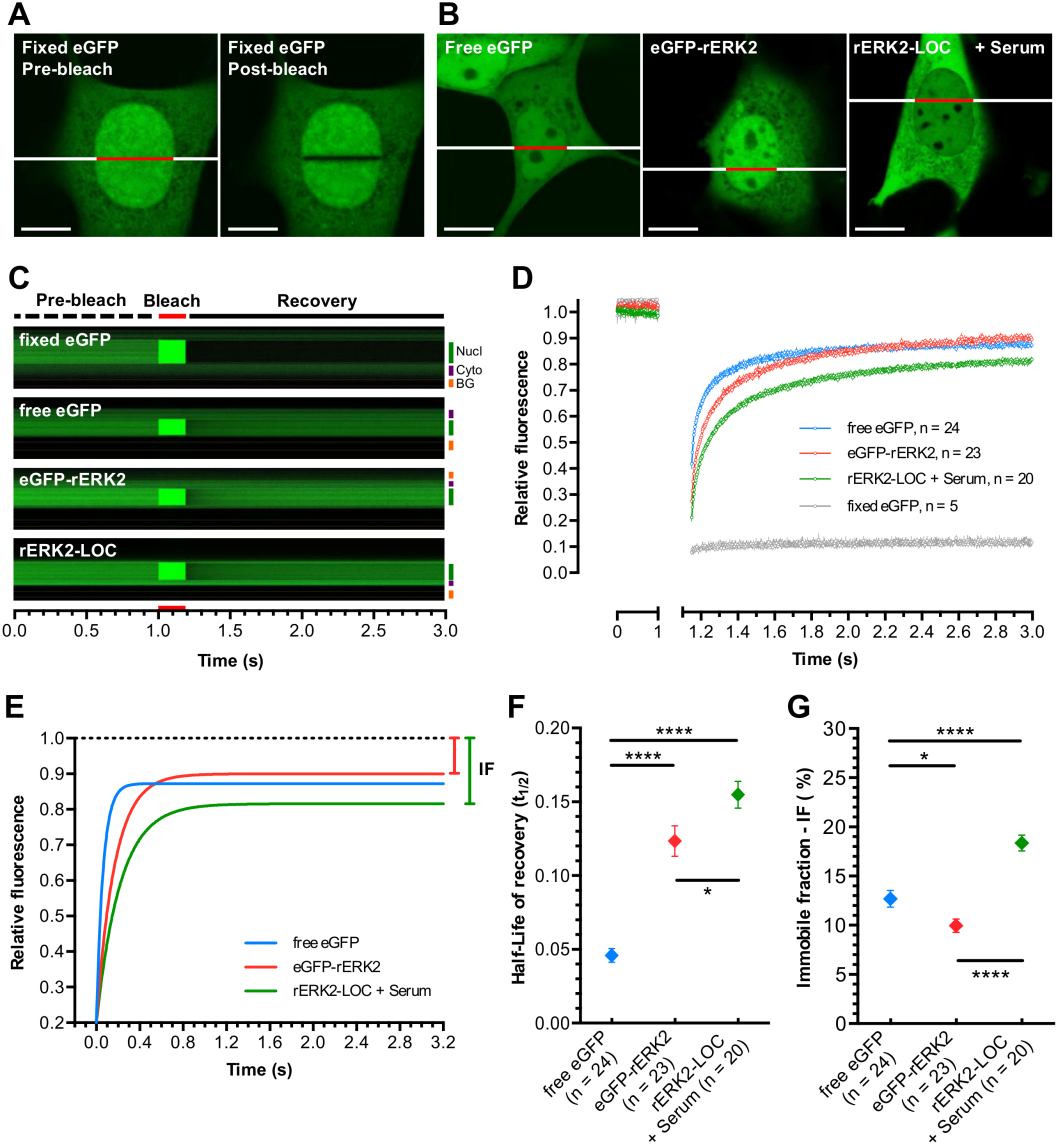
Mobility of rERK2-LOC measured by high-speed FRAP. **(A)** eGFP-transfected NIH-3T3 cells were fixed. Individual living cells were imaged as described in the Materials and Methods section. Image sequences before (left) and after (right) photobleaching are shown. Scale bars: 10 μm. **(B)** NIH-3T3 cells were transfected with eGFP (left), eGFP-rERK2 (middle) or rERK2-LOC (right) and then serum starved for 24 h. Cells overexpressing rERK2-LOC were stimulated with serum to trigger its nuclear translocation. Bleached ROI correspond to the red lines drawn across the nuclei. Scale bars: 10 μm. **(C)** Representative kymograms (xt) of fluorescence intensity measured along the line (both red and white) across the selected cells for each experimental condition over-time are shown, indicating the FRAP measurement sequence: pre-bleach of 1 s (broken dark line), bleach of 150 ms (red lines) and post-bleach of 2 s (solid dark line). Correction for overall bleaching effects was applied. Nucl: nucleus (green line), Cyto: cytoplasm (purple line), BG: background (yellow line). **(D-E)** Curves of cumulative fluorescence recovery over time for fixed eGFP (grey curve), free eGFP (blue curve), overexpressed eGFP-rERK2 (red curve) and rERK2-LOC after serum stimulation (green curve, 8 min after serum stimulation) were normalized **(D)** and fitted **(E)**. **(F-G)** Average half-life of recovery (t_1/2_) and immobile fraction (IF) calculation for cells serum-starved for 24 h and overexpressing free eGFP (blue symbol) or eGFP-rERK2 (red symbol), and serum-stimulated cells overexpressing rERK2-LOC (green symbol, 8 min after serum stimulation). At least two independent experiments were performed. The number of individual cells used for each condition is indicated above each symbol. Statistical significance was determined by a two-tailed unpaired *t*-test (ns, no significant; *, ≤ 0.05; ****, ≤ 0.0001).

To finalize our characterization and validation in living cells, we determined mobility of rERK2-LOC in living NIH-3T3 cells in comparison to that of overexpressed eGFP-rERK2 or eGFP alone using high-speed FRAP measurements. Fixed NIH3T3 cells expressing eGFP were used to calibrate our imaging setup (Fig 4A). Based on rERK2-LOC dynamics in serum-starved NIH-3T3 cells after FGF4 stimulation (Fig 3E), only serum-stimulated cells with rERK2-LOC accumulating in the nucleus were imaged and compared to cells overexpressing free eGFP or eGFP-rERK2 (Fig 4B and 4C).

Comparative analysis of cumulative fluorescence recovery curves showed that nuclear free eGFP (blue curve) retained very high mobility, reflecting the passive diffusion of the fluorescent protein throughout the cell (Fig 4D). The fluorescence recovery curve of overexpressed eGFP-rERK2 (red curve) mimicked that of free eGFP, as previously reported [29]. In contrast, after serum stimulation and accumulation of rERK2-LOC in the nucleus (t = 8 min after stimulation), fluorescence recovery of rERK2-LOC (green curve) indicated a marked reduction of mobility in the nucleus, which contrasts with a previous study reporting no difference in mobility measurements between overexpressed free eGFP and eGFP-rERK2 [26].

Next, fluorescence recovery curves were fitted to a one-phase exponential-association equation (Fig 4E) and the recovery process was characterized by the half-life of fluorescence recovery (t_1/2_) to accurately describe and compare protein mobility (Fig 4F). In comparison with the extremely high mobility of free eGFP (t_1/2_ = 0.046 s ± 0.005, n = 24), the half-life recovery of overexpressed eGFP-rERK2 *versus* rERK2-LOC were 0.123 s ± 0.010 (n = 23) and 0.155 s ± 0.009 (n = 20), respectively (p = 0.03). We did not observe a more significant difference between eGFP-rERK2 and rERK2-LOC, indicating that overexpressed eGFP-rERK2 may still bind slightly to nuclear partners. We also calculated the percentage of the immobile fraction (IF) (Fig 4G) defined by the value between the complete fluorescence recovery asymptote and the pre-bleach value being equal to 1 (Fig 4E, black dotted line). The corresponding values for free eGFP and overexpressed eGFP-rERK2 were 12.68% ± 0.85 (n = 24) and 9.95% ± 0.68 (n = 23), respectively (*p* ≤ 0.05). This was clearly significantly different from the value obtained with rERK2-LOC (IF = 18.37% ± 0.80, n = 20, *p* ≤ 0.0001). Thus, despite very rapid ERK2 shuttling to and from the nucleus, we detected, as previously reported [29], a significantly slower mobility and turnover of rERK2-LOC in the nucleus of stimulated cells compared to overexpressed eGFP-rERK2.

Additional FRAP experiments were next performed to assess changes in the mobility of rERK2-LOC between the cytoplasm and the nucleus of serum-starved NIH-3T3 cells before and after serum stimulation (S1 Fig). Following the same experimental protocol, a stripe across the nucleus and the cytoplasm of the same cell was bleached a few seconds apart (Figure A in S1 Fig). As shown in Figures B-D in S1 Fig, the immobile fraction of rERK2-LOC was significantly reduced in the cytoplasm of serum-stimulated cells (IF = 6.36% ± 0.99, n = 12) in comparison to that of rERK2-LOC in the cytoplasm of serum-starved cells (IF = 14.00% ± 2.70, n = 9, *p* ≤ 0.05), demonstrating the dissociation of a pool of rERK2-LOC from its cytoplasmic partners upon stimulation. Interestingly, the immobile fraction of rERK2-LOC in the nuclei of serum-stimulated cells (IF = 14.83% ± 1.26, n = 12) was significantly larger from that of rERK2-LOC in the cytoplasm of the same analyzed cells (*p* ≤ 0.0001). We observed also similar immobile fractions of rERK2-LOC in the nuclei of serum-stimulated cells and the cytoplasm of serum-starved cells, suggesting that rERK2-LOC binds to nuclear and cytoplasmic scaffolds/anchors, respectively. Collectively, the data obtained with equimolar expression of eGFP-rERK2 and mMEK1 are consistent with previous studies using different strategies to report ERK1/2 dynamics in living cells [29,30]. In contrast to these studies, no stringent imaging conditions were required, making our approach compatible with long-term functional monitoring of ERK2 dynamics in living cells.

### Spatiotemporal subcellular distribution of xERK2-LOC in *Xenopus* laevis embryo

**Fig 5:**
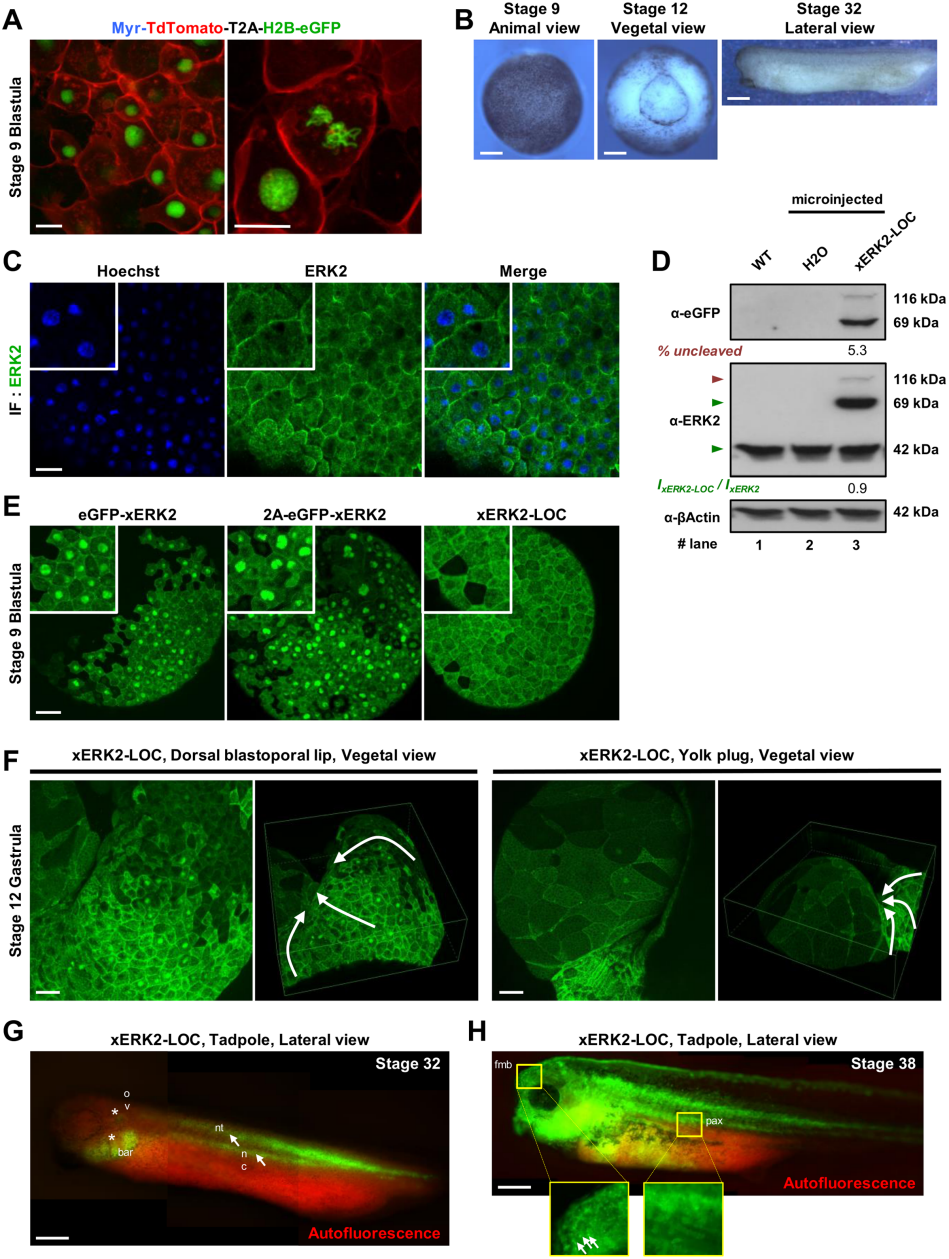
Spatiotemporal subcellular distribution of xERK2-LOC in living *Xenopus laevis* embryo. **(A)** Embryos were injected with 500 ng of Myr-TdTomato-T2A-Histone2B-GFP mRNA. Maximum-intensity projection of a z-stack of 40 confocal images with a z-step of 0.59 μm is shown at 40X (left, scale bar: 10 μm) and at 63X magnification (right, scale bar: 20 μm). Pictures were merged to visualize Myr-mCherry at the plasma membrane and H2B-eGFP in the nucleus. **(B)** xERK2-LOC subcellular distribution was visualized at several stages of *Xenopus laevis* development (stage 9 blastula, stage 12 blastula and stage 32 tadpole). Scale bar: 200 μm. **(C)** Stage 9 embryos were fixed and processed for immunofluorescence with antibody against total ERK2 (green) and stained for DNA with Hoechst (blue) as described in the Materials and Methods section. Scale bar: 100 μm. **(D)** Protein extracts were prepared from uninjected (WT, lane 1), H_2_O injected (lane 2) and xERL2-LOC overexpressing embryos (lane 3) and immunoblotted with antibodies against GFP (top panel), ERK2 (middle panel) and β-actin (bottom panel). The percentage of uncleaved xERK2-LOC was measured by densitometry and is shown below the top panel in lane 3. The levels of overexpressed xERK2-LOC relative to endogenous ERK2 (green triangles, middle) are indicated below the blot as ***I***_***xERK2-LOC***_ ***/ I***_*xERK2*._ **(E)** Embryos were injected with 500 ng of eGFP-xERK2, 2A-eGFP-xERK2 (control) or xERK2-LOC mRNA. Projections of at least 60 confocal 0.7-μm sections of animal cells at stage 9 blastula are shown. Higher magnification images of representative subcellular distributions of xERK2 are shown in white squares (top left). Scale bar: 150 μm. **(F)** Monitoring of xERK2-LOC subcellular distribution in the cells of the dorsal blastoporal lip (left panel) and the yolk plug (right panel) in stage-12 gastrula. Projection and 3D reconstruction of z-series of 108 confocal 1.50-μm sections (left panel) and 86 confocal 1.00-μm sections (right panel) are shown. White arrows indicate the trajectories of the cells leading to a progressive internalization of the yolk plug. Scale bar: 150 μm. **(G-H**) Several images from different viewpoints were recorded and combined to create a whole image of the developing embryos expressing xERK2-LOC, head to the left, at stage 32 (**G**) and stage 38 (**H**) tadpoles. The spatiotemporal localization of xERK2-LOC (green) in the embryonic structures, enhanced by autofluorescence of embryo and yolk (red signal), corresponds to notochord (nc), neural tube (nt) (both white arrows), otic vesicle (ov) and branchial arch region (bar) (both white asterisks) **(G)**. Higher magnification of the forebrain-midbrain boundary (fmb) and para-axial structures (pax) are shown in yellow squares **(H)**. Small white arrows indicate xERK2-LOC nuclear accumulation in several cells of the forebrain-midbrain boundary. Scale bar: 500 μm. At least two independent experiments were performed from animal caps, fixed embryos, or live embryos, and at least ten embryos were imaged. Biochemical data are representative of at least two independent experiments.

After fully characterizing and successfully validating the faithful reporting of ERK2 dynamics by our T2A-mediated coexpression system in living cells, we tested our reporter rERK2-LOC in a relevant multicellular model organism. In *Xenopus laevis* embryos, FGF signaling plays a crucial role in the formation of mesoderm [41] and particularly in maintenance of the mesoderm through a feedback loop that involves MAPK/ERK2 cascade-mediated stabilization of Brachyury expression [42–44]. Several studies used a specific antibody against activated ERK1/2 in whole *Xenopus laevis* embryos at different stages of development. Immunohistochemical analysis showed strong activation of ERK2 in whole-mount embryos at the end of gastrulation around the dorsal lip of blastopore [45,46]. To gain insight into ERK2 localization in relation to spatiotemporal patterns of ERK2 activation at different stages of development (Fig 5B), we further employed our 2A-mediated coexpression approach. To test whether the T2A peptide functions in *Xenopus laevis* embryos, we microinjected mRNA from the original plasmid pMyr-TdTomato-T2A-H2B-eGFP (Fig 2A, #1) into embryos at the one-cell stage. Maximum-intensity projection showed that in stage 8 embryos H2B-eGFP and Myr-TdTomato were present exclusively in the nucleus and at the plasma membrane, respectively, as reported in other model organisms [47,48] (Fig 5A). To determine the subcellular distribution of endogenous xERK2, we immunostained fixed, whole-mount, stage 8 embryos with anti-ERK2 antibody (Fig 5C). Overlay of anti-ERK2 and Hoechst staining revealed that most xERK2 was localized in the cytoplasm of ectodermal cells.

To explore xERK2 dynamics in living embryos, we microinjected embryos at the one-cell stage with mRNA encoding eGFP-xERK2, 2A-eGFP-xERK2 without xMEK1 sequence as a control, or xMEK1-2A-eGFP-xERK2 (xERK2-LOC). Given that ERK2 can be activated by mechanical stress or wounding [46], live imaging of intact embryos was performed, while preserving ectodermal tissue integrity (Fig 5E). Maximum-intensity projection revealed nuclear accumulation of eGFP-xERK2 and 2A-eGFP-xERK2. In contrast, expression of xERK2-LOC resulted in a more homogenous distribution of the kinase within blastomeres, with a slight tendency towards the cytoplasm, reminiscent of the immuno-localization of endogenous ERK2 in fixed embryos (Fig 5C). In parallel, we assessed xERK2-LOC protein expression levels in embryos at stage 8 by western blot analysis (Fig 5D) using both anti-GFP (upper panel) and anti-ERK2 (middle panel) antibodies. The proportion of uncleaved polypeptide was slightly lower in *Xenopus laevis* embryos (5.3%) in comparison to cultured NIH-3T3 cells (Fig 2D, upper panel).

At the gastrula stage (stage 11), we investigated xERK2-LOC subcellular distribution in embryonic cells of the dorsal lip of the blastopore region, where FGF signaling is known to activate ERK1/2 pathway. In line with this information, we observed a patch of ectodermal cells just above the dorsal lip of the blastopore exhibiting a strong accumulation of xERK2-LOC in the nucleus (Fig 5F, left panel and S3 Movie). These observations are in agreement with the previously reported immuno-localization of activated di-phosphorylated ERK2 [46]. Finally, a nuclear localization of xERK2-LOC was seen in large endodermal cells of the yolk plug (Fig 5F, right panel and S4 Movie), although no activation of ERK2 had been detected by immunohistochemistry [46].

Since no toxicity was observed and embryos developed normally, we explored the localization of xERK2-LOC at later developmental stages in living embryos. At the early tadpole stage (stage 32), GFP fluorescence was detected mainly in the neural tube, the notochord, and the somites (white arrows), as well as in the otic vesicle and the branchial arch region (white asterisks) (Fig 5G). Red fluorescence due to known autofluorescence of *Xenopus laevis* embryos was advantageously used to achieve optimum contrast for accurate localization of xERK2-LOC in embryonic structures. At stage 38, when the *Xenopus laevis* tadpole becomes transparent, xERK2-LOC was widespread in the head region and the para-axial structures (Fig 5H and S5 Movie). Remarkably, xERK2-LOC accumulated in the nuclei of a small patch of cells in the forebrain-midbrain boundary but not in para-axial structures (Fig 5H and S5 Movie). Once again, our results highlighted the faithful subcellular distribution of xERK2 provided by xERK2-LOC in comparison to immuno-localized activated di-phosphorylated ERK2 in the *Xenopus laevis* embryo.

## Discussion

The MAPK/ERK1/2 pathway plays an important role in many cellular processes: cell proliferation, migration, differentiation, and even cell death [14,49–51]. Aside from the activity of ERK1/2, its subcellular localization is instrumental in signal integration in the cell fate decision [8,52,53]. Many approaches have been used to monitor ERK1/2 dynamics in living cells, but some of them do not localize the kinase of interest correctly in non-stimulated cells [24,26]. Moreover, they are often laborious and unsuitable for long-term imaging [29], or they are time consuming because transgenic cell lines have to be generated [30]. We overcame these limitations by designing a novel molecular tool, ERK2-LOC. Characterization and validation of the tool in living cells and tissue showed that ERK2-LOC is functional, faithful, easy to use, and biologically relevant.

Various studies used ERK2 tagged with GFP-like fluorescent proteins to monitor the spatiotemporal localization of ERK2 in individual living cells. However, these studies disregarded the predominantly nuclear localization of eGFP-rERK2 in resting cells [16,23–25] (Fig 1). It has been known for a long time that disruption of the MEK/ERK balance disturbs ERK2 localization [12,15]. Aside from favoring conditions where a low expression level of eGFP-rERK2 was managed [29] (Fig 1D), this problem was solved by co-expressing mMEK1, thereby restoring the proper cytoplasmic localization of overexpressed eGFP-rERK2 in serum-starved cultures without stimulation [26,27]. This results in a more controlled MEK/ERK ratio in transfected cells (Fig 2B, upper panel). Given that expression levels of co-transfected mCherry-MEK1 and eGFP-ERK2 in the same cell cannot be controlled due to the limitations of co-transfection techniques [26] (Fig 2B, upper panel), ERK2 subcellular distribution throughout the cell is necessarily affected. From this observation, it became obvious that proper quantification of ERK2 dynamics in response to specific stimuli requires a robust system for reliable coexpression at the single cell level. While multiple heterologous proteins can be coexpressed in living cells by different approaches, such as use of the Internal Ribosomal Entry Site (IRES) sequence and use of bidirectional or multiple promoters in the same plasmid, these systems suffer from problems related to coexpression efficiency [54,55]. A more promising approach described as a 2A-mediated coexpression system (for review [33,48,56]), was used in our study. 2A-linked proteins have been efficiently expressed *in vitro* in a wide variety of cultured eukaryotic cells and embryonic stem cells, and even *in vivo* in embryos and whole organisms [47,48] but never reported in *Xenopus laevis* model. While no protein degradation or side effects of premature termination of translation have been reported [57], previous work described variability in the 2A peptide-mediated cleavage, depending on the choice of 2A peptide and the cellular model [48]. Further support for the 2A strategy is found in previous studies demonstrating robust equimolar coexpression of this approach in studies of the molecular interactions of G-coupled proteins [55] and T-cell development in CD3-deficient mice [58]. Although we used an optimized peptide (see Materials and Methods), a slight difference in cleavage efficiencies between NIH-3T3 cells and an embryonic cell system was noted. The efficiency ranged from 91.1% (Fig 2D) in NIH3T3 cells to 94.7% in *Xenopus* embryos (Fig 5D); the presence of an uncleaved MEK1/ERK2 polypeptide could affect ERK2 functions in both model systems. Actually, MEK1—ERK2 fusion polypeptide was reported to produce a constitutively active fusion ERK2 in the absence of upstream signaling [59]. In this context, a mutated form of MEK1 (nuclear export/activity region) in fusion with ERK2 was able to induce PC12 differentiation and NIH3T3 transformation. Wild type and mutated MEK1-ERK2 fusions had no effect on the activity of endogenous ERK1/2 [59]. Interestingly, immunofluorescence results showed that the subcellular localization of the mutated MEK1—ERK2 fusion protein was nuclear, while that of the wild type was essentially cytoplasmic. A more efficient 2A derived peptide, such as P2A, might be used to alleviate system perturbation linked to uncleaved MEK1—ERK2 polypeptide [48].

Recent studies quantified the MEK/ERK ratio in different cellular contexts by biochemical approaches [60,61]. Both proteins are in the micromolar range, but the reported MEK/ERK ratios in HeLa cells are considerably different: 3.1/2.1 [62], 1.4/0.96 [60] and 1/10 [61], as well as in PC12 cells, 0.6/1.25 [63] and 0.68/0.26 [64]. They also varied depending on the cell type, ranging from 1 in *Xenopus laevis* oocyte, Cos7 and Rat1 cells, to about 2 in CHO, 208F, and PC12 cells, and up to almost 13 in NIH3T3 cells [40,60,61]. Recently the role of ERK2 has been emphasized. In a murine system, ERK2^-/-^ embryonic lethality was attributed to failure of placenta and trophoblastic development [65,66], while ERK1^-/-^ embryos are viable and fertile but have problems in thymic development. In the same line of thought, knockdown of ERK2 in *zebrafish* model prevents epiboly and the blastula to gastrula transition, while ERK1 knockdown provokes subtle defects in the embryogenesis [67]. Moreover, the reported 4/1 ERK2/ERK1 ratio in NIH-3T3 cells in both relative and activated forms was proposed to explain the preeminent role of ERK2 in cellular functions over that of ERK1 [68]. In the *Xenopus laevis* model, the maternally inherited ERK2 isoform is important for oocyte maturation and the MEK/ERK ratio is 1:1 [60,69]. In all cellular models, ratios were determined in systems at equilibrium. In our approach, one can only assume that co-expression generated equimolar ERK2 and MEK1 concentrations, but these were not quantified in our experimental systems at equilibrium. Therefore, we settled on a consensual 1:1 co-expression ratio of MEK1/ERK2 based on the following considerations. First, these studies quantified the pool of ERK1/2 and MEK1/2 rather than distinct isoforms. Second, various MEK/ERK ratios have been reported for the same cell type. Third, it is technically difficult to express 13 times more rERK2 than mMEK1 proteins in living NIH3T3 cells. Fourth, we intended to use our reporter in the *Xenopus laevis* model system. Indeed, our purpose here was foremost to counter-balance eGFP-rERK2 overexpression with identical amounts of its partner MEK1. We did not intend to reproduce a mammalian expression system shadowing that of the cellular model system or even a synthetic network, but rather to faithfully mimic the subcellular distribution of the endogenous ERK2 for functional monitoring purposes.

Our approach resulted in the intended disruption of the initial MEK/ERK balance by co- overexpression of ERK2 and MEK1 in equimolar proportions at the single cell level. We show that it did not disturb ERK2 dynamics in living cells (Fig 3E) or embryos (Fig 5G and 5H). Although ERK2 is the only ERK isoform expressed in *Xenopus* embryos until mid-blastula transition, a two-fold increase in the proportion of total xERK2 (Fig 5D) and overexpression of xMEK1 *via* our xERK2-LOC reporter did not alter embryonic development (Fig 5G and 5H). In addition, overexpression of eGFP-xERK2 was not toxic to *Xenopus* embryos, indicating that artefacts in eGFP-xERK2 nuclear localization were not detrimental to ongoing cellular programs at these developmental stages (data not shown). Specific GFP fluorescence signals were detected at later stages (late gastrula, tadpole), pointing to the long half-life of exogenous xERK2 protein in these embryos. We conclude that our strategy is a non-invasive method for assessing functional ERK2 dynamics in living embryos during early *Xenopus laevis* embryogenesis. We were well aware of the uncoupling functions of ERK, but we used ERK2 localization in *Xenopus* as a surrogate for ERK activation. Any disruptive effects of xERK2-LOC on the signaling network will be further characterized in the future.

In contrast to overexpressed eGFP-rERK2, the dynamics of ERK2-LOC was faithful under our experimental conditions. Visualization of rERK2-LOC was actually enhanced at the single living cell level, as shown by fluorescence microscopy (Fig 3B and 3D). Depending on the treatment, ERK2-LOC nuclear translocation or cytoplasmic retention was readily visible (Fig 3A) and faithfully matched that of the endogenous pattern. This was not the case for overexpressed eGFP-rERK2 even in U0126 pre-treated cells (in the presence or absence of serum or FGF4). Cells accumulated overexpressed eGFP-rERK2 in the nucleus independently of MEK1-mediated TEY-phosphorylation (Fig 3A, middle image row, U0126 treatments), pointing-out the limit of eGFP-rERK2 over-expression and questioning the consequences and relevance of MEK1 phosphorylation of the overexpressed eGFP-rERK2 in this experimental context. The marked absence of a fluorescence signal in the nuclei of rERK2-LOC transfected cells in non-stimulated conditions (Fig 2B, lower panel and Fig 3A, bottom row left) resembles the endogenous situation (Fig 3A, top row left). This prompted us to assess the role of overexpressed MEK1, and we showed that the increased amount of MEK1 in the cellular system was responsible for retention of the pool of endogenous ERK2 in the cytoplasm (Fig 2C).

Protein over-expression is bound to affect signaling networks and cellular functions. Using Fig 2B as an example, FGF increases the CI for endogenous ERK1/2 by about 50% but increases it about ten-fold for the rERK2-LOC reporter. The balance of ERK2 binding to MEK1 *versus* other interacting proteins, such as anchors, scaffold, activators and effectors, is likely influenced. ERK2 activation of targets may well be increased, and overexpressed MEK1 likely also influences endogenous ERK2 and the binding of exogenous ERK2 to DNA and microtubules. To prevent over-expression from perturbing the spatio-temporal aspects of the signaling pathways, recently developed alternative approaches could be implemented. Nowadays, based on directed genome editing technology by Clustered Regularly Interspaced Short Palindromic Repeats (CRISPR) [70], endogenous proteins can be knocked in to insert fluorescent proteins. However, based on our experience, two conditions should be met to make long-term functional imaging feasible: the expression level of the protein of interest should be sufficiently high, and fluorescent proteins with a high quantum yield (brightness) should be used.

Quantitative analysis of rERK2-LOC after different treatments (Fig 3B) also faithfully shadowed that of endogenous ERK2, with comparable kinetic in NIH3T3 cells (Fig 3F and S1 Movie) and in HeLa cells (S2 Movie). This enhanced translocation was seen in time-lapse experiments, in which NIH3T3 cells expressing rERK2-LOC were monitored every 2 min before and after FGF4 treatment (Fig 3F). Based on the calculated concentration index, an initial nuclear burst of rERK2-LOC was visible, peaking between 4 to 8 minutes after FGF4 induction, as expected [29]. With regards to over-expression driven perturbations of the signaling network, several explanations can be proposed for the subsequent CI decrease. Nuclear anchors saturation and the presence of exogenous MEK1 in ERK2 export from the nucleus could be responsible. However the expected sustained activation profile of ERK2 [29] has been maintained since CI did not decrease to initial baseline level.

Concerning ERK2 diffusion, the significantly slower mobility of rERK2-LOC compared to overexpressed eGFP-rERK2 in FRAP experiments (Fig 4D) indicates the stimulus-dependent binding of ERK2 to specific nuclear targets. So, saturation of ERK2-binding sites due to overexpression of eGFP-rERK2 without sufficient amount of MEK1 around altered the shuttling and resulted in accumulation of the kinase in the nucleus, where eGFP-rERK2 behaved as a free monomer. However the slight difference in diffusion of overexpressed eGFP-rERK2 (Fig 4D) could indicate that it might still bind slightly to nuclear partners. Our results using rERK2-LOC unambiguously showed a decrease of ERK2 mobility in the nucleus, demonstrating that equimolar coexpression of mMEK1 counterbalances the overexpression of eGFP-rERK2 and thus prevents saturation of the limited ERK2 nuclear binding sites. This result is at odds with a previous study [26] that reported no difference in mobility between overexpressed free eGFP and eGFP-rERK2, and which was attributed to the use of cells with strong overexpression [29]. Because of the high ERK2 nuclear concentration, detection of eGFP-rERK2 nuclear binding upon stimulation of the pathway was not possible in their experimental settings.

As described in previous studies, mitogenic stimulation triggers rapid entry of ERK2 into the nucleus followed by massive nuclear accumulation of ERK2 several hours after the stimulation. On the other hand, non-mitogenic signals trigger only the initial translocation of ERK2 [21,71]. The characteristic mitogenic response was observed in NIH-3T3 cells transfected with rERK2-LOC, whereas an abnormally brief nuclear localization of ERK2 was generally associated with uncontrolled coexpression of MEK1/ERK2 [26,27,30]. Indeed, rERK2-LOC subcellular distribution was identical to that of endogenous ERK1/2 [21], with progressive nuclear accumulation 1 h and 2 h after either serum or FGF4 stimulation. It was reported that the late nuclear accumulation of ERK2 requires nuclear anchors such as MKP1 and MKP2, the expression of which is induced by ERK1/2 signaling [21,22,71]. ERK2-mediated phosphorylation of MKPs triggers inactivation and nuclear retention of ERK2 through high-affinity interactions, limiting access to activated MEK1 in the cytoplasm. Consistent with previous studies, this late accumulation of ERK2 in the nucleus is uncoupled from MEK1-dependent TEY-phosphorylation of ERK2 [20,22]. Thus, since rERK2-LOC subcellular distribution matched endogenous ERK2 localization over time, we suggest that our rERK2-LOC reporter is regulated in the same way as the endogenous ERK2 by the endogenous regulatory proteins of the ERK1/2 signaling pathway. In addition, recent findings identified a similar mechanism for uncoupling TEY-phosphorylation from ERK2 nuclear localization at the early phase of the stimulation [20]. This uncoupling mechanism is not explainable by the sole expression of specific nuclear anchors and relies on a Casein Kinase 2-dependent SPS-phosphorylation in the kinase insert domain of ERK2 that is independent of ERK2 activation [72]. In *Xenopus laevis* embryo, spatiotemporal distribution of xERK2-LOC coincided with that of phosphorylated ERK2 in the region around the blastopore at stage 12, where an increase of ERK2 activation occurs, as well as at late gastrula and tadpole stages [46] (Fig 5E, left panel, Fig 5G and 5H and S3 Movie). In addition, we also found xERK2-LOC in the nuclei of large cells of the yolk plug at stage 12 (Fig 5F, right panel and S4 Movie), although no activation of ERK2 was previously detected by immunohistochemistry [46]. These findings shed light on the importance of closely correlating ERK1/2 activation to its subcellular localization to determine cell fate and assess the involvement of specific spatiotemporal regulators of the ERK1/2 pathway. Considering the kinase-independent functions of ERK2 that have been reported both in the cytoplasm and in the nucleus [73,74], this has become particularly relevant.

## Conclusion

In this study, limitations in eGFP-tagged ERK2 expression were solved by using a T2A “self-cleaving” peptide in bicistronic plasmids. Previous studies have shown that the cleavage efficiency of T2A is much higher than that of other 2A sequences [47,48]. So we fused the T2A sequence in frame between MEK1 and ERK2. The 2A peptide strategy enabled equimolar coexpression of MEK1 and ERK2 and restored the localization dynamics of ERK2. More importantly, we show that the expression pattern of the coexpressed proteins was consistent among the transfected cells. We confirmed the functionality of rERK2-LOC by using several biochemical approaches. Upon stimulation, rERK2-LOC rapidly translocated into the nucleus, but its translocation was blocked by MEK1/2 inhibition. Fast-FRAP experiments in the nucleus and in the cytoplasm revealed a differential diffusion of rERK2-LOC, depending on its activation state and its subcellular localization. Given the stability of T2A-linked protein coexpression, we coexpressed MEK1 and eGFP-xERK2 in *Xenopus laevis* embryos to monitor xERK2 localization at different embryonic developmental stages. This is the first report on the subcellular localization of xERK2 in living embryos. Our ERK2-LOC reporters could be used in conjunction with ERK1/2 activity measurements [60,75,76] in several biological systems to assess whether pharmacological inhibitors affect specifically ERK1/2 activity and/or ERK2 subcellular distribution [7]. Finally, this 2A-mediated coexpression system is versatile and makes it possible to build on existing reporters by adding coding sequences from other genes (Raf, KSR, PEA-15) that are relevant to the regulation of the ERK1/2 signaling pathway. Taken together, our study has revealed that 2A-mediated coexpression of eGFP-ERK2 and MEK1 is a reliable and user-friendly strategy to faithfully monitor ERK2 in living cells and in a whole organism.

## Supporting Information

S1 File Supplementary material, plasmid constructs. (PDF)

S1 Fig Comparison of rERK2-LOC mobility in the nucleus and cytoplasm of NIH-3T3 by high-speed FRAP measurements. **(A)** NIH-3T3 cells were transfected with rERK2-LOC and serum-starved for 24 h. Bleaching was first performed in the cytoplasm of non-stimulated cells, and in both the nucleus and the cytoplasm of the same cell after serum stimulation along the red lines drawn (left panel). Representative kymograms (xt) of fluorescence intensity measured along the lines (both red and white) across the selected cells for each experimental condition over time are shown (right panel). Scale bar: 10 μm. **(B-C)** Curves of cumulative fluorescence recovery over time for rERK2-LOC in resting cell cytoplasm (blue curve), and in cytoplasm (green curve) and nucleus (red curve) 8 min after serum stimulation were normalized **(B)** and fitted **(C)**. **(D)** Immobile fractions (IF) were calculated for all conditions (corresponding color symbols). The number of photobleached cells is indicated above each symbol. Statistical significance was determined by a two-tailed unpaired *t*-test (ns, no significant; *, ≤ 0.05; ****, ≤ 0.0001).
(PDF)

S1 Movie rERK2-LOC spatiotemporal localization in serum-starved NIH-3T3 cells after FGF4 stimulation. (MP4)

S2 Movie rERK2-LOC spatiotemporal localization in serum-starved HeLa cells after hEGF stimulation. (MP4)

S3 Movie xERK2-LOC subcellular distribution in a living *Xenopus laevis* embryo at the dorsal lip of the blastopore. The movie shows a vegetal view of the embryo (stage 12, late gastrula) and is made from 108 confocal z-planes using a 1.50-μm step size between sections. The confocal z-series 3D reconstruction of the dorsal lip of blastopore shows the accumulation of rERK2-LOC in the nuclei of blastoporal cells located in the push inward area.
(MP4)

S4 Movie xERK2-LOC subcellular distribution in a living *Xenopus laevis* embryo at the yolk plug. The movie shows a vegetal view of the embryo (stage 12, late gastrula) overexpressing xERK2-LOC and is made from 86 confocal z-planes using a 1.00-μm step size between sections. The confocal z-series 3D reconstruction of the yolk plug shows the accumulation of rERK2-LOC in the nuclei of large endodermal cells.
(MP4)

S5 Movie Imaging of xERK2-LOC in a whole living *Xenopus laevis* stage 38 tadpole. The embryo, head to the left, shows substantial nuclear accumulation of xERK2-LOC in the cells of the forebrain-midbrain boundary.
(MP4)
