# Supplementary material for: Novel Reporter for Faithful Monitoring of ERK2 Dynamics in Living Cells and Model Organisms
Source: PLoS One. 2015 Oct 30;10(10):e0140924. doi: 10.1371/journal.pone.0140924 (PMC4627772; doi:10.1371/journal.pone.0140924)
Supplement: S1 File — (PDF) [file pone.0140924.s001.pdf]

Novel reporter for faithful monitoring of ERK2 dynamics in living cells and model organisms.

François Sipieter, Benjamin Cappe, Mariano Gonzalez Pisfil, Corentin Spriet, Jean-François Bodart, Katia Cailliau-Maggio, Peter Vandenabeele, Laurent Héliot, Franck B. Riquet.

Plasmid Constructs

pCS2-xMEK1-2A-GFP-xERK2: The *Xenopus laevis* ERK2 sequence (xERK2) was amplified by polymerase chain reaction (PCR) using the forward primer xERK2.AgeI-BamHI-F (*Age*I and *Bam*HI sites underlined; start codon in bold), and the reverse primer xERK2.XhoI-R, (*Xho*I site underlined; stop codon in bold). To amplify eGFP from pEGFP-N1 (Clontech, USA), forward primer eGFP.AscI-F (*Asc*I site underlined) and reverse primer eGFP.NheI-R (*Nhe*I site underlined) without stop codon were then designed. xERK2 and eGFP PCR products were purified, digested and ligated sequentially into the pCS2-7G backbone at the *Age*I–*Xho*I and *Asc*I–*Nhe*I sites, respectively, to create the intermediary vector pCS2-eGFP-7G-xERK2. We adopted a cassette-cloning strategy using the complementary oligonucleotides T2A.AscI-F and T2A.AscI-R to generate a double-stranded cassette containing the 2A peptide sequence derived from the *Thosea asigna* virus (italic) and two unique restriction sites, *Spe*I and *Cla*I (underlined). The resulting cassette flanked by *Asc*I overhangs was then ligated into the pCS2-eGFP-7G-xERK2 vector at the *Asc*I site to create the control vector pCS2-2A-eGFP-7G-xERK2. The *Xenopus laevis* MEK1 sequence (xMEK1) was then obtained by two-step PCR from five stage-8 blastula embryos. Poly(A)+ RNA was reverse transcribed into cDNA, and after first-strand synthesis, the cDNA was used as a template for the PCR step. xMEK1 was amplified by PCR using the forward primer xMEK1.SpeI-F and the reverse primer xMEK1.ClaI-R, containing *Spe*I and *Cla*I sites, respectively (underlined). The PCR product was ligated into the *Spe*I and *Cla*I sites in the T2A cassette to produce the final expression vector pCS2-xMEK1-2A-eGFP-7G-xERK2. Note that the Gly-Ser-Gly (GSG) linker between MEK1 and the T2A sequences was maintained to optimize cleavage efficiency [1,2].

pCS2-mMEK1-2A-GFP-rERK2: The eGFP-rERK2 sequence from peGFP-C1-rERK2 was obtained by digestion with *Nhe*I and *Bam*HI restriction enzymes. eGFP-rERK2 was then purified and ligated into the *Nhe*I and *Bam*HI sites in the pCS2-7G backbone to create the expression vector pCS2-eGFP-rERK2. The *Mus musculus* MEK1 (mMEK1) sequence was obtained using the procedure described above for xMEK1, using 100 ng of total RNA extracted from NIH-3T3 cells. mMEK1 was amplified by PCR using the forward primer mMEK1.SpeI-F and the reverse primer mMEK1.ClaI-R, containing *Spe*I and *Cla*I sites,

respectively (underlined). The PCR product was ligated into the *Spe*I and *Cla*I sites in the previously constructed pCS2-2A-eGFP-7G-xERK2 to produce the intermediary vector pCS2-mMEK1-2A-eGFP-7G-xERK2. The mMEK1-2A fragment was excised from the intermediary vector using *Asc*I, purified, and ligated in frame to the corresponding restriction site in linearized pCS2-eGFP-rERK2 to create the final expression vector pCS2-mMEK1-2A-eGFP-rERK2.

pCS2-mCherry-mMEK1-2A-GFP-rERK2: The peGFP-N1 vector was digested with *Age*I and *Not*I to remove the eGFP sequence, and then filled in with Klenow and re-ligated. The resulting vector was cut with *Nhe*I and a cassette containing a 7-glycine linker (italic) and two unique restriction sites, *Age*I and *Bsr*GI (underlined), were inserted in the *Nhe*I site, producing pClontech-N1-7G. The forward and reverse complementary oligonucleotides used to create the cassette were Backbone-7G-F2 and Backbone-7G-R2. The mCherry sequence from pmCherry-N1 vector was then subcloned into the *Age*I and *Bsr*GI restriction sites of pClontech-N1-7G to generate the pClontech-7G-mCherry intermediary vector. pClontech-N1-7G-mCherry and pCS2-mMEK1-2A-eGFP-rERK2 were digested with *Nhe*I and *Spe*I, respectively, and incubated with Mung Bean Nuclease to create blunt-ends extremities. The resulting 7G-mCherry fragment was ligated into pCS2-mMEK1-2A-eGFP-rERK2, producing the final expression vector pCS2-mCherry-mMEK1-2A-eGFP-rERK2.

pmCherry-7G-mMEK1: The pmCherry-C1 vector was cut with *Bsp*EI and *Xho*I. Complementary oligonucleotides Backbone-7G-F3 and Backbone-7G-R3 were annealed to each other to generate a double-stranded cassette containing a 7-glycine linker (italic) and two unique restriction sites, *Spe*I and *Asc*I (underlined). The cassette flanked by *Bsp*EI and *Xho*I overhangs was then ligated into the pmCherry-C1 at *Bsp*EI and *Xho*I sites to create the pmCherry-C1-7G intermediary vector. mMEK1 was amplified by PCR using the forward primer mMEK1.SpeI-F2, incorporating a *Spe*I site (underlined) and a stop codon (bold), and the reverse primer mMEK1.AscI-R (*Asc*I site underlined). The PCR product was ligated into pmCherry-C1-7G at the *Spe*I and *Asc*I sites, producing the final expression vector pmCherry-C1-7G-mMEK1.

1. Minskaia E, Ryan MD. Protein coexpression using FMDV 2A: effect of “linker” residues. Biomed Res Int. 2013;2013: 291730. doi:10.1155/2013/291730

2. Yu KK, Aguilar K, Tsai J, Galimidi R, Gnanapragasam P, Yang L, et al. Use of mutated self-cleaving 2A peptides as a molecular rheostat to direct simultaneous formation of membrane and secreted anti-HIV immunoglobulins. PLoS One. 2012;7: e50438. doi:10.1371/journal.pone.0050438
