# Supplementary figures and images for: Novel Reporter for Faithful Monitoring of ERK2 Dynamics in Living Cells and Model Organisms
Source: PLoS One. 2015 Oct 30;10(10):e0140924. doi: 10.1371/journal.pone.0140924 (PMC4627772; doi:10.1371/journal.pone.0140924)

**A**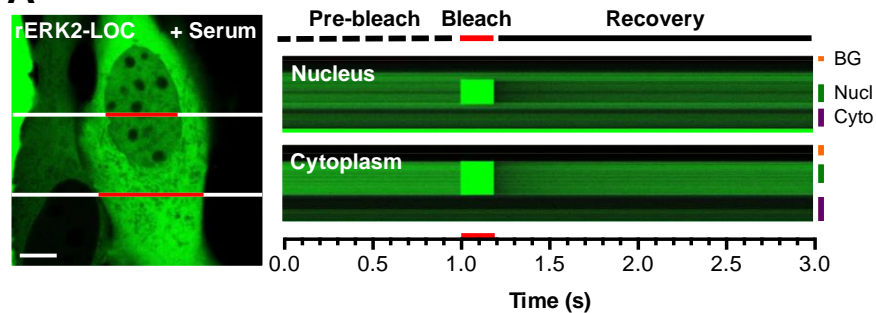**B**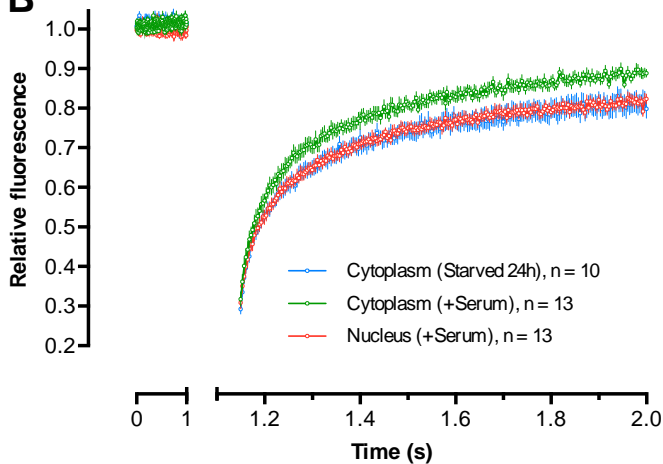**C**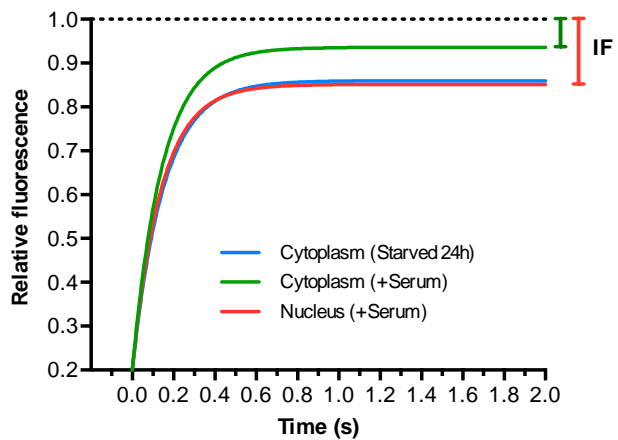**D**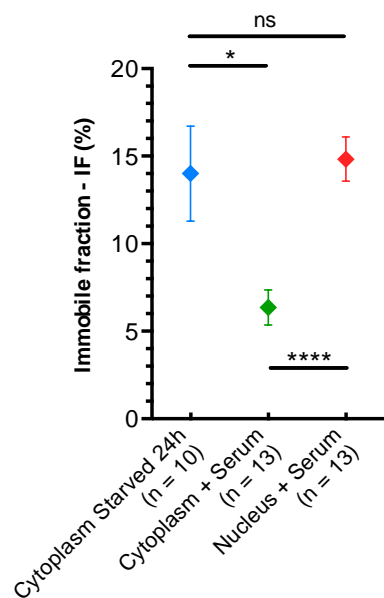

Supplement: S1 Fig — (A) NIH-3T3 cells were transfected with rERK2-LOC and serum-starved for 24 h. Bleaching was first performed in the cytoplasm of non-stimulated cells, and in both the nucleus and the cytoplasm of the same cell after serum stimulation along the red lines drawn (left panel). Representative kymograms (xt) of fluorescence intensity measured along the lines (both red and white) across the selected cells for each experimental condition over time are shown (right panel). Scale bar: 10 μm. (B-C) Curves of cumulative fluorescence recovery over time for rERK2-LOC in resting cell cytoplasm (blue curve), and in cytoplasm (green curve) and nucleus (red curve) 8 min after serum stimulation were normalized (B) and fitted (C). (D) Immobile fractions (IF) were calculated for all conditions (corresponding color symbols). The number of photobleached cells is indicated above each symbol. Statistical significance was determined by a two-tailed unpaired t-test (ns, no significant; *, ≤ 0.05; ****, ≤ 0.0001). (PDF) [file pone.0140924.s002.pdf]
